# The Photosynthetic Apparatus and Its Regulation in the Aerobic Gammaproteobacterium *Congregibacter litoralis* gen. nov., sp. nov

**DOI:** 10.1371/journal.pone.0004866

**Published:** 2009-03-16

**Authors:** Stefan Spring, Heinrich Lünsdorf, Bernhard M. Fuchs, Brian J. Tindall

**Affiliations:** 1 Deutsche Sammlung von Mikroorganismen und Zellkulturen, Braunschweig, Germany; 2 Helmholtz Zentrum für Infektionsforschung, Braunschweig, Germany; 3 Max-Planck-Institut für Marine Mikrobiologie, Bremen, Germany; National Institute on Aging, United States of America

## Abstract

**Background:**

There is accumulating evidence that in some marine environments aerobic bacteriochlorophyll *a*-producing bacteria represent a significant part of the microbial population. The interaction of photosynthesis and carbon metabolism in these interesting bacteria is still largely unknown and requires further investigation in order to estimate their contribution to the marine carbon cycle.

**Methodology/Principal Findings:**

Here, we analyzed the structure, composition and regulation of the photosynthetic apparatus in the obligately aerobic marine gammaproteobacterium KT71^T^. Photoheterotrophically grown cells were characterized by a poorly developed lamellar intracytoplasmic membrane system, a type 1 light-harvesting antenna complex and a photosynthetic reaction center associated with a tetraheme cytochrome *c*. The only photosynthetic pigments produced were bacteriochlorophyll *a* and spirilloxanthin. Under semiaerobic conditions KT71^T^ cells expressing a photosynthetic apparatus showed a light-dependent increase of growth yield in the range of 1.3–2.5 fold. The expression level of the photosynthetic apparatus depended largely on the utilized substrate, the intermediary carbon metabolism and oxygen tension. In addition, pigment synthesis was strongly influenced by light, with blue light exerting the most significant effect, implicating that proteins containing a BLUF domain may be involved in regulation of the photosynthetic apparatus. Several phenotypic traits in KT71^T^ could be identified that correlated with the assumed redox state of growing cells and thus could be used to monitor the cellular redox state under various incubation conditions.

**Conclusions/Significance:**

In a hypothetical model that explains the regulation of the photosynthetic apparatus in strain KT71^T^ we propose that the expression of photosynthesis genes depends on the cellular redox state and is maximal under conditions that allow a balanced membrane redox state. So far, bacteria capable of an obligately aerobic, photosynthetic metabolism constitute a unique phenotype within the class Gammaproteobacteria, so that it is justified to propose a new genus and species, Congregibacter litoralis gen. nov, sp. nov., represented by the type strain KT71^T^ ( = DSM 17192^T^ = NBRC 104960^T^).

## Introduction

The oceans harbor a huge population of diverse microorganisms that are involved in the global cycling of carbon. A study of the major players participating in the marine carbon cycle is important to estimate the evolving capacity of oceans as carbon dioxide sink. In the last few years numerous cultivation-independent studies have been published that illustrate the abundance, diversity and distribution of aerobic bacteriochlorophyll *a*-producing bacteria in marine environments [*e.g.* 1]–[3]. However, many questions regarding their evolution and importance for the carbon cycle remain unanswered. For instance, the environmental factors that determine expression of bacteriochlorophyll genes in aerobic bacteria are only partly understood [4], [5]. The impact of light on the growth yield of most species is also still unknown, although the number of aerobic bacteriochlorophyll-containing bacteria available in pure culture and thus accessible to a phenotypic characterization is steadily increasing [6]. On the other hand, it is widely accepted that environmental data solely indicating a presence of bacteriochlorophyll-containing bacteria in the aerobic euphotic zone of oceans are not sufficient to make reliable estimates of the extent of light-driven carbon assimilation. Furthermore, the available physiological studies on marine aerobic anoxygenic photosynthetic bacteria are restricted to isolates belonging to distinct clades of the *Alphaproteobacteria* [for review see 7], so that the derived assumptions may be biased. Hence, a detailed analysis of diverse isolates representing abundant clades of marine aerobic bacteriochlorophyll-containing bacteria is essential and a prerequisite for a deeper understanding of this interesting phenotype. Only a profound knowledge of this type of metabolism will allow us to evaluate various evolutionary models that currently try to explain the origin and function of anoxygenic photosynthesis in aerobic marine bacteria [*e.g.* 8], [9].

**Figure 1:**
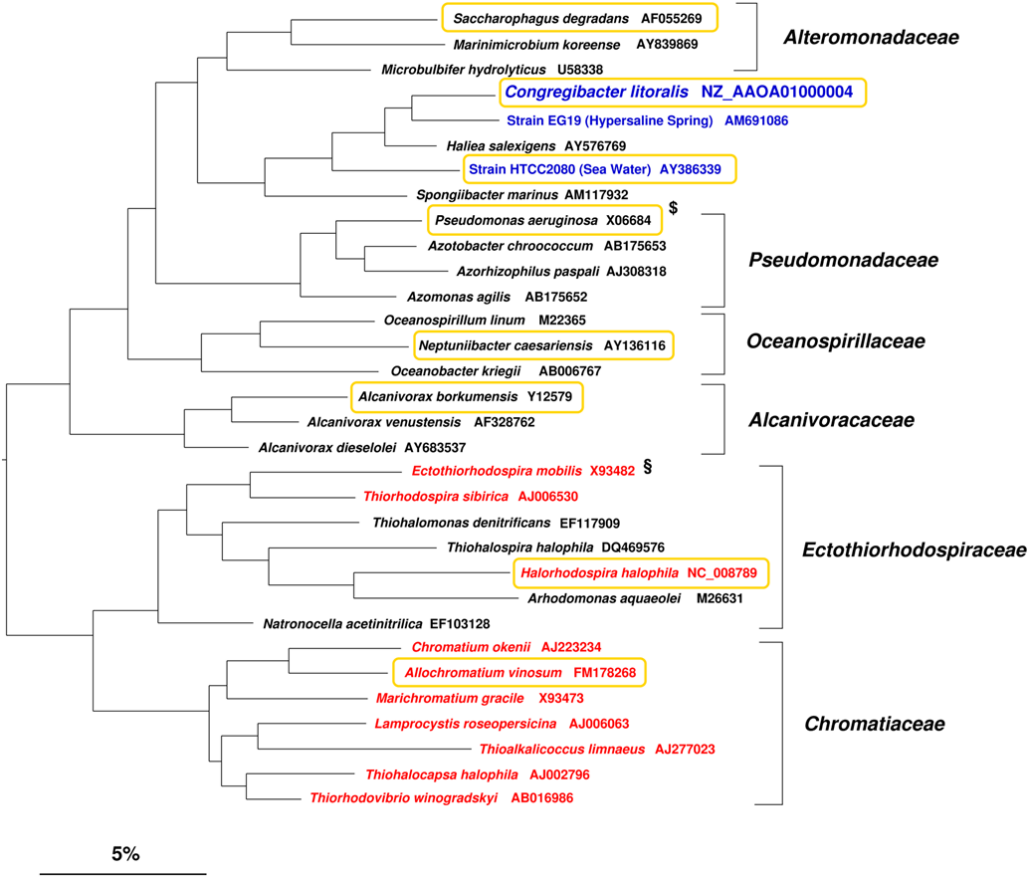
Phylogenetic dendrogram illustrating the position of *Congregibacter litoralis* KT71^T^ among cultured representatives of the class *Gammaproteobacteria*. Unless noted otherwise almost-complete 16S rRNA gene sequences of type strains were used for tree reconstruction. The sequence of *Escherichia coli* (X80725) was used as outgroup (not shown). Phylogenetic analyses were based on the alignment included in the SILVA database (release 95) [89] and trees were reconstructed using distance matrix (neighbor-joining), parsimony and maximum-likelihood algorithms using programs implemented in the ARB software package [90]. The dendrogram shown was reconstructed using a maximum-likelihood program (fastDNAml) and represents a branching order that was also frequently obtained when alternative programs were used. Representatives of anaerobic anoxygenic photosynthetic bacteria are emphasized with red lettering, whereas aerobic bacteriochlorophyll *a*-producing bacteria are shown in blue lettering. The availability of a genome sequence is indicated by an orange frame. Notes: $, several genome sequences of *Pseudomonas aeruginosa* strains are currently available, but none was obtained from the type strain. §, until now no 16S rRNA gene sequence of the type strain of *Ectothiorhodospira mobilis* is available so that the sequence of DSM 4180 was chosen.

We have chosen strain KT71^T^ as a model organism to study aerobic anoxygenic photosynthesis, because it represents a hitherto unrecognized group of marine bacteriochlorophyll-containing gammaproteobacteria [2]. A preliminary description of strain KT71^T^ based on a draft genome sequence has been published and it was shown that this isolate represents a novel taxon tentatively named “*Congregibacter litoralis*” [10]. Phylogenetically, KT71^T^ is affiliated to a major cluster of environmental 16S rRNA gene sequences that were retrieved mainly from marine habitats. This phylogenetic group has been designated OM60 by Rappé et al. [11] or NOR5 by Eilers et al. [12] and belongs to the class *Gammaproteobacteria*. The phylogenetic position of KT71^T^ among cultured representatives of non-photosynthetic and photosynthetic gammaproteobacteria is shown in Figure 1. Currently, the most closely related classified species are *Haliea salexigens* [13] and *Spongiibacter marinus* [14], which have both been described as non-photosynthetic bacteria. Approaches using *in situ* hybridization of whole cells with specific oligonucleotide probes revealed that members of this cluster are metabolically active in marine environments [12], [15] and thus can play a major role in the littoral and euphotic zones of the oceans. The presence of bacteriochlorophyll genes in aerobic gammaproteobacteria other than KT71^T^ has been demonstrated by metagenomic [16] or cultivation-based studies (strains HTCC2080 [17] and EG19 [18]). Thus, besides the well-known alphaproteobacterial *Roseobacter* clade [19], [20] a second phylogenetic group of aerobic anoxygenic photosynthetic bacteria in marine environments is emerging, of which KT71^T^ is one cultured representative. In the course of a detailed characterization of this isolate, it was found that the synthesis of photosynthetic pigments was occasionally induced following a transfer from complex medium to defined medium containing only a single carbon source. Among various carbon sources tested malate had the most stimulating effect on the production of photosynthetic pigments. Based on this finding photoheterotrophically growing cultures of KT71^T^ could be obtained, which were used for an extensive analysis of the composition and regulation of the photosynthetic apparatus in this bacterium. The results obtained in this study allow the proposal of a metabolic model that helps to understand the function of anoxygenic photosynthesis in KT71^T^. Furthermore, the proposed concept may provide some clues to the evolution, distribution and ecological importance of aerobic anoxygenic photosynthetic bacteria in ocean waters.

## Results

### Characteristics of the Photosynthetic Apparatus

#### Ultrastructure and intracytoplasmic membrane system

**Figure 2:**
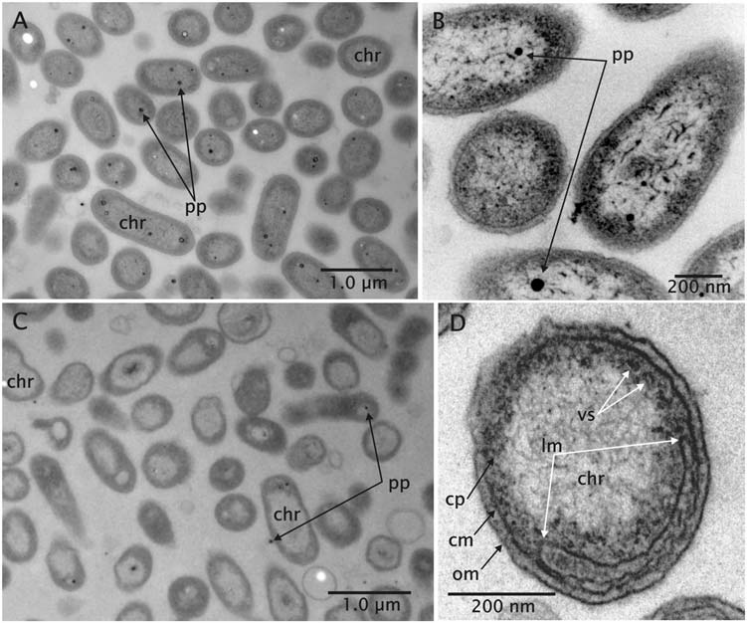
Ultrastructure and intracytoplasmic membranes. (A,B) Chemoheterotrophic growth. (C,D) Photoheterotrophic growth. Abbreviations: chr, chromosome; pp, polyphosphate inclusion; vs, vesicle; om, outer membrane; lm, lamellar membrane invagination; cm, cytoplasmic membrane; cp, cytoplasm. (A,C) 90 nm ultrathin sectioned cells after osmium and uranium pre- and lead citrate-uranylacetate poststaining. (B,D) Untreated 35 nm sectioned cells as reversed prints of HCI images.

Ultrathin sections of KT71^T^ cells revealed a typical Gram-negative cell wall with an inner cytoplasmic membrane and an outer membrane. The nucleoide was obviously concentrated in a central region of the cell that is less electron dense compared to the surrounding cytoplasm. Small electron dense particles that were frequently observed in ultrathin sections could be identified as polyphosphate inclusions by electron energy-loss spectroscopy. Interestingly, these inclusions could be detected mainly in cells grown under chemoheterotrophic conditions (Figure 2A, B).

In cells that were cultured under conditions allowing optimal expression of photosynthetic pigments the development of an intracytoplasmic membrane system was detected by transmission electron microscopy of ultrathin sections (Figure 2D). The membrane invaginations were of the lamellar type and adhere to the inner surface of the cytoplasmic membrane, similar to those observed in *Rhodopseudomonas palustris* [21]. However, in contrast to *Rhodopseudomonas palustris* intracytoplasmic membranes were only poorly developed in KT71^T^ and no thylacoidal membrane stacks were formed. In chemoheterotrophically grown cells that did not express photosynthetic pigments a similar differentiation of the cytoplasmic membrane was hardly detectable (Figure 2B).

#### Structure and size of the reaction center - antenna complex

**Figure 3:**
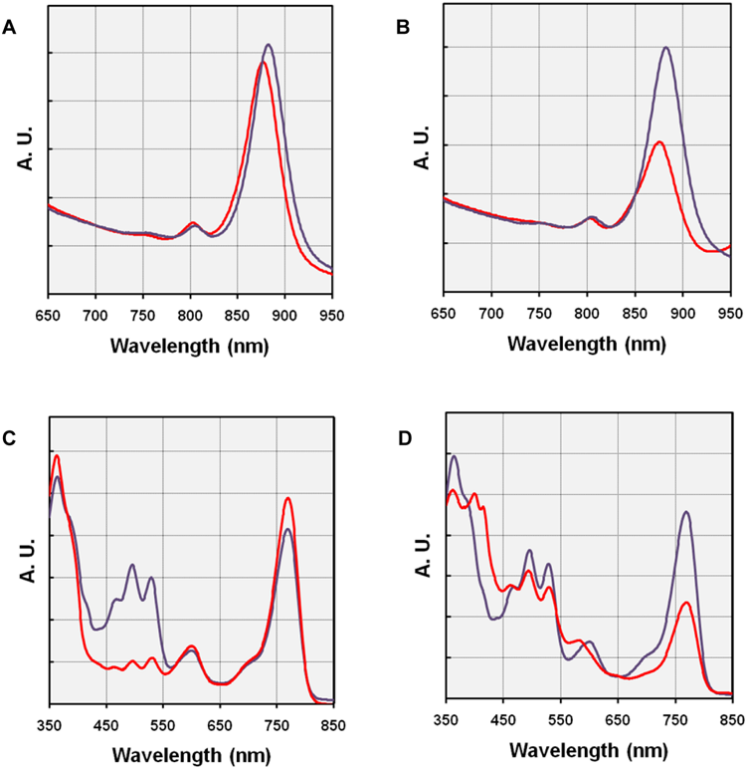
Structure and composition of the light-harvesting apparatus. (A,B) Whole cells spectra of KT71^T^ cultures grown with 5 mM DL-malate as substrate under semiaerobic conditions (12 vol% initial oxygen concentration) in the light (A) and with 2 mM succinate under microaerobic conditions (6 vol% initial oxygen concentration) in darkness (B). The blue lines represent a whole cells spectrum of a *R. rubrum* culture incubated anaerobically in the light. It is included as reference and has been manually adjusted to the same absorbance at 700 nm. (C,D) Spectra of acetone/methanol extracts obtained from KT71^T^ cells grown in semiaerobic SMFC medium (12 vol% initial oxygen concentration) under illumination with blue light (C) and grown with 2 mM pyruvate in microaerobic medium (6 vol% initial oxygen concentration) in darkness (D). The spectrum of an acetone/methanol extract from an aged culture of *R. rubrum* (blue lines) is included as reference and has been manually adjusted to the same absorbance at 650 nm. A.U., absorbance (arbitrary units).

Whole cells absorption-spectra of photoheterotrophically grown cultures of KT71^T^ were determined and compared with spectra obtained with cultures of *Rhodospirillum rubrum* DSM 467^T^ incubated anaerobically in the light. The photosynthetic apparatus of *R. rubrum* comprises a reaction center (RC) enclosed by a circular light-harvesting antenna complex (LH1), but no secondary (*i.e.* peripheral) light-harvesting (LH2) antenna complex [22]. As shown in Figure 3A the *in vivo* spectra determined from both strains were nearly identical. A minor difference is that the two major absorption bands of strain KT71^T^ in the near-infrared region were located at 802 and 876 nm and thus are blue-shifted in respect to *R. rubrum* DSM 467^T^ (peaks located at 804 and 882 nm). This indicates that the photosynthetic apparatus of strain KT71^T^ comprises only one type of light-harvesting antenna complex (LH1, peak at 876 nm) and a photosynthetic reaction center (RC, peak at 802 nm) both forming a core complex. Even upon incubation under conditions that are unfavorable for photosynthesis (*e.g.* in the dark) no indication for a peripheral LH2 complex in KT71^T^ cells could be detected, which would give rise to a more intense peak around 800 nm and a shoulder or an additional peak at 820–850 nm in the respective spectra. Consequently, it can be assumed that no significant amount of the LH2 complex is expressed in KT71^T^ cells under laboratory growth conditions, although in the genome of this strain the genes *pucA* (annotated open reading frame (ORF): KT71_03077) and *pucB* (annotated ORF: KT71_03072) were found that encode structural polypeptides forming the LH2 complex [10].

Suboptimal conditions for photosynthesis and pigment production caused a significant decrease of the LH1 complex size in KT71^T^ cells (Figure 3B). This assumption is based on the observation of a smaller peak ratio between the long-wavelength (Q_y_) absorption bands of the LH1 antenna bacteriochlorophyll *a* (BChl *a*) at 876 nm and the reaction center accessory BChl *a* at 802 nm compared to spectra of KT71^T^ cells grown under optimal conditions for photosynthesis or cells of *R. rubrum* DSM 467^T^, which are known to express a LH1-RC core complex with a relatively constant stoichiometry of 32 BChl *a* molecules per reaction center [23]. Recent studies applying high resolution electron microscopy or atomic force microscopy on photosynthetic membranes support the view of a LH1 complex that is variable in size and shape, at least in some bacteria [24], [25]. Alternatively, the variable absorption of the LH1 antenna complex in KT71^T^ cells may be independent of the actual size but due to perturbations in the BChl *a* environment leading to a lower LH1-BChl extinction coefficient.

#### Pigment composition and stoichiometry

Identification of the major pigments in photoheterotrophically grown KT71^T^ cells was done by UV/visible spectroscopy of acetone/methanol extracts prepared from wet cell pellets. The results essentially confirm the finding of Fuchs et al. [10], who used HPLC in combination with a photodiode array detector to identify the photosynthetic pigments BChl *a* and spirilloxanthin in KT71^T^ cells. In this study BChl *a* was identified in crude extracts by characteristic peaks at 363, 600, and 770 nm and spirilloxanthin by peaks at 465, 495 and 529 nm. The values obtained coincide well with published data for BChl *a* [26] and spirilloxanthin [27]. For comparison aged cultures of *R. rubrum* DSM 467^T^, which are known to contain BChl *a* and spirilloxanthin as major photosynthetic pigments, were extracted applying the same protocol as used for KT71^T^. Under optimal conditions for photosynthesis the UV/visible spectra indicate an almost identical pigment composition for both strains. However, in contrast to *R. rubrum* the pigment stoichiometry in KT71^T^ was highly variable with BChl *a* to spirilloxanthin ratios ranging from about 2 to 10 (Figure 3C, D). Besides BChl *a* and spirilloxanthin no other photosynthetic pigments could be identified in KT71^T^ cells grown under various conditions. However, sometimes an additional pigment type could be detected under incubation conditions that lead to a repression of photosynthetic pigment synthesis. For instance, cell suspensions of cultures that were incubated in the dark showed frequently a bright yellow-orange pigmentation and an additional type of compound displaying peaks between 400 and 420 nm became apparent in acetone/methanol extracts (Figure 3D). It is unlikely that these compounds represent carotenoids due to the highly variable spectral fine structure. Extraction of freeze-dried KT71^T^ cells with chloroform/methanol and separation of extracts by thin layer chromatography (data not shown) resulted in the development of a distinct band comprised of yellow to orange-red compounds that could be easily distinguished from photosynthetic pigments, which had a much lower polarity. Following separation by thin layer chromatography the unknown pigments were again dissolved in acetone/methanol to determine UV/visible spectra. The spectra obtained displayed a broad absorption maximum in the visible range between 350 and 570 nm thus explaining the unusual high absorbance values in this region in the spectrum shown in Figure 3D. Therefore, under certain conditions production of such polar pigments may lead to an overestimation of the spirilloxanthin concentration by the spectral reconstruction method.

The amount of BChl *a* and spirilloxanthin produced per cellular dry weight depended largely on the incubation conditions. Under optimal conditions for photoheterotrophic growth A_880 nm_/A_660 nm_ values of 0.94–1.05 were obtained in cultures of KT71^T^. In acetone/methanol extracts of the harvested cells the following pigment concentrations were determined (15 samples, standard deviation in parentheses): 3.02 (±0.42) nmol BChl *a*/mg (dry weight, dw) and 0.81 (±0.19) nmol spirilloxanthin/mg (dw). The cellular dry weight was calculated from the A_660 nm_ value of the harvested culture. Accordingly, the BChl *a* to spirilloxanthin ratio averages 3.7 under optimal conditions for pigment expression. A quantification of photosynthetic pigments in extracts of photoheterotrophically grown *R. rubrum* cultures (A_880 nm_/A_660 nm_ values of 1.22–1.44) prepared by the same method resulted in the following values (5 samples): 4.48 (±0.46) nmol BChl *a*/mg (dw) and 1.84 (±0.21) nmol spirilloxanthin/mg (dw). The dry weight of the material extracted was calculated from the A_680 nm_ value of the harvested culture using a multiplication factor of 0.87 mg [28]. As aged cultures of *R. rubrum* were used for pigment extraction spirilloxanthin represented the prevailing carotenoid in the respective spectra (Figure 3C, D), so that the molar extinction coefficient of spirilloxanthin could be used for the estimation of the carotenoid abundance. The calculated average BChl *a*/spirilloxanthin ratio in whole cells of *R. rubrum* was 2.4 and hence considerably lower than in KT71^T^ cells. Within the boundaries of experimental error the values obtained for the type strain of *R. rubrum* are in good agreement with published data about this strain [*e.g.* 29], thereby indicating that the values determined for KT71^T^ should be also valid. Consequently, KT71^T^ represents an exception to the common assumption that aerobic anoxygenic photosynthetic bacteria are in general characterized by a low bacteriochlorophyll to carotenoid ratio [7].

#### Cytochrome composition, quinones and polar lipids

**Figure 4:**
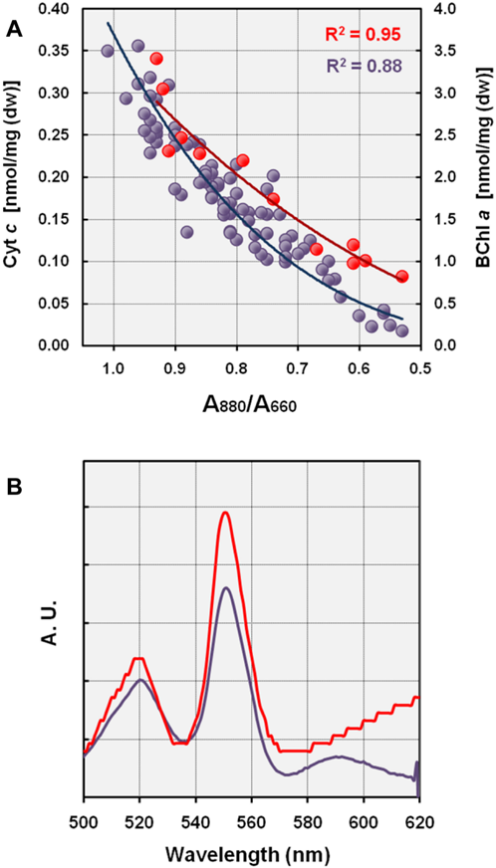
Cytochrome composition in photosynthetically active and unpigmented cells. (A) Correlation of bacteriochlorophyll *a* (blue circles) and high-potential heme *c* (red circles) abundance with the expression of the photosynthetic apparatus. The amount of cytochrome *c* was determined by redox difference spectroscopy of solubilized cell extracts. The concentration of bacteriochlorophyll *a* in acetone/methanol extracts of wet cell pellets was estimated by the spectral reconstruction method of van der Rest and Gingras [26]. Various expression levels of the photosynthetic apparatus in KT71^T^ cells were achieved by transferring photoheterotrophically grown cultures to microaerobic or semiaerobic media containing different carbon sources. (B) Dithionite-reduced *minus* ferricyanide-oxidized redox difference spectra of pyridine hemochromes extracted from photoheterotrophically grown cells (red line) and cells grown chemoheterotrophically in SYPG medium (blue line) under microaerobic conditions. Bands at 550 and 588 nm indicate pyridine hemochromes *c* and *a*, respectively. Pyridine hemochrome *b* is recognized by a shoulder at 557 nm.

Redox carrier proteins and quinones represent an essential part of the photosynthetic apparatus in all anoxygenic photosynthetic proteobacteria. Open reading frames proposed to represent genes encoding proteins that could play a role in photosynthetic and respiratory electron transport are listed in Table S1. As periplasmic cytochrome types, which are known to participate in the electron transport chain, only the types *c*_4_ and *c*_5_ were found, whereas no cytochromes *c*_2_ or *c*_8_ were annotated. A gene coding for a reaction center cytochrome *c* subunit is located within the photosynthesis genes superoperon (PGS). In addition, two genes were identified encoding different high-potential iron-sulfur proteins (HiPIPs), which are known to play a role in respiration and photosynthesis [30]. Genes for an ubiquinol-cytochrome *c*-oxidoreductase (cytochrome *bc_1_* complex) and three putative terminal oxidases were also found. Hence, all elements necessary for the operation of a Q-cycle mode of energy conservation could be localized in the KT71^T^ draft genome. In order to identify compounds of the electron transport chain that are essential for photosynthesis the cytochrome (cyt) composition in KT71^T^ was analyzed in photoheterotrophically grown cells (5 mM DL-malate as substrate) and compared with the composition in unpigmented cells grown chemoheterotrophically in various media. We used N,N-dimethyldodecylamine-N-oxide (LDAO) for the extraction and solubilization of cytochromes from whole cells, because this requires only a small amount of biomass. It should be noted that the solubilization of certain membrane-bound cytochromes requires rather harsh methods like the extraction of membranes with butanol [31]. Hence, it is possible that not all cytochrome types were solubilized completely by using the mild detergent LDAO, but instead remained partly in the insoluble material that was separated from the supernatant. Redox difference spectroscopy was used to analyze the expression of cytochromes in batch cultures that were incubated under various initial oxygen concentrations ranging from 3 to 21 vol%. Common characteristics of the obtained redox difference spectra were a Soret or gamma band at 422–426 nm, a beta band located at 521–525 nm and an alpha peak at 552–553 nm (Figure S1A). These spectral features are characteristic for *c*-type cytochromes [32], which therefore represent the dominating fraction of cytochromes in KT71^T^ cells. By far the highest expression levels of cyt *c* were found in cells expressing a photosynthetic apparatus. The appearance of redox difference spectra derived from solubilized extracts of these cells was quite similar to those from chromatophore membranes of phototrophic bacteria expressing a reaction center tetraheme cytochrome, like for instance *Allochromatium vinosum* [33] or *Rhodoferax fermentans* [34]. The intensities of the cyt *c* peak (Δε_552-538_) in ascorbate-reduced *minus* ferricyanide-oxidized difference spectra of KT71^T^ cells correlated well with the expression of photosynthetic pigments. This effect was independent of the substrate utilized and confirms the presence of a reaction center cytochrome *c* subunit. In Figure 4A the correlation of the expression level of the photosynthetic apparatus with the abundance of high-potential cyt *c* and BChl *a* is shown. The data obtained could be fitted to single potential curves with coefficient of determination (R^2^) values of 0.95 for cyt *c* (12 samples) and 0.88 for BChl *a* (83 samples). At maximal expression of the photosynthetic apparatus the ratio between BChl *a* and high-potential cyt *c* approximates 10∶1. In fully pigmented KT71^T^ cells only about 75% of solubilized cytochromes *c* were reduced by ascorbate compared to a complete reduction with dithionite, so that for total cyt *c* the ratio would be 10∶1.25. Assuming that the solubilized heme *c* in photoheterotrophically grown cells is predominantly bound to the tetraheme cytochrome the ratio would be 10 BChl *a* to 0.3125 reaction center cytochrome *c* or 32∶1, which is the theoretically expected value for a photosynthetic reaction center embedded in a LH1 complex containing 28 BChl *a* molecules.

In addition to cyt *c*, cytochromes containing heme *a* or *b* as prosthetic groups were identified. Low-potential cytochromes *b* were of low abundance and probably overshadowed by the more abundant *c*-type cytochromes in dithionite-reduced *minus* ferricyanide-oxidized difference spectra. Cytochromes containing heme *a* became apparent in redox difference spectra by a shoulder of the Soret band at 445 nm along with a broad alpha peak at 601–604 nm. This assumption was confirmed by a distinct band at 588 nm in pyridine hemochrome difference spectra (Figure 4B). It can be deduced from the annotated genome sequence of KT71^T^ that heme *a* is most likely bound to a cytochrome *caa_3_* oxidase (Table S1). Surprisingly, only 50% of the total heme *a* was reduced by ascorbate, which could be explained by a resting form of the oxidase that is transferred into the fully active (or pulsed) form by reduction with dithionite. A similar effect was previously described for a novel *aa_3_*-type oxidase from *Pseudomonas aeruginosa* [35]. Interestingly, the lowest expression level of cytochromes containing heme *a* were repeatedly found in photoheterotrophically growing cells, whereas in cells grown in SYPG medium under microaerobic conditions a maximal expression level of cytochrome *aa_3_* was observed and estimated to be 0.03 nmol/mg (dw).

Lipid soluble isoprenoid quinones are involved in electron transfer and transport of protons across the cytoplasmic membrane during photophosphorylation. Chemical analysis of the quinone composition in KT71^T^ cells grown chemoheterotrophically in SYPG medium as well as photoheterotrophically with 5 mM DL-malate as substrate revealed ubiquinone 8 as the sole respiratory lipoquinone, so that it can be assumed that this quinone type is also present in the photosynthetic reaction center. Likewise, the composition of polar lipids, which were mainly represented by phosphatidylethanolamine and phosphatidylglycerol along with an unidentified phospholipid, remained unchanged under chemoheterotrophic and photoheterotrophic growth conditions.

#### Energy yield of aerobic photosynthesis

**Figure 5:**
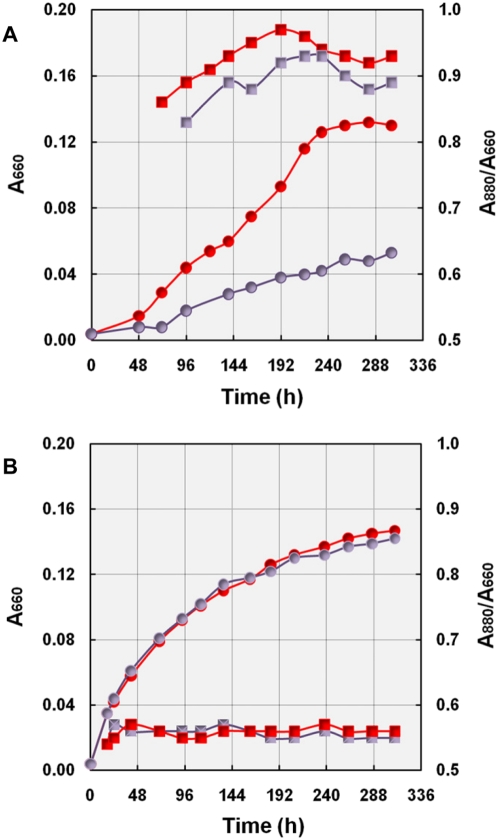
Comparison of photoheterotrophic and chemoheterotrophic growth. Growth curves of strain KT71^T^ under microaerobic conditions (6 vol% initial oxygen concentration) with 2 mM L-malate as carbon source (A) or in half-strength SYPG medium (B). Circles represent A_660 nm_ values and squares A_880 nm_/A_660 nm_ values. Red and blue symbols indicate incubation in light and darkness, respectively.

To demonstrate the generation of metabolically useful energy from light, growth curves of KT71^T^ cells expressing various amounts of photosynthetic pigments were determined in light and darkness. A 40 W tungsten filament incandescent lamp was used for illumination resulting in a light intensity of about 1400 lux (equivalent to 28 µE m^−2^ s^−1^). The effect of light on growth was quantified by comparing dry weights from total biomass produced under light and dark incubation. In addition, the production of photosynthetic pigments in growing cells was followed by recording A_880 nm_/A_660 nm_ values. In Figure 5 representative growth curves of KT71^T^ cultures incubated in two different media are shown. KT71^T^ cells growing in defined medium with L-malate synthesized photosynthetic pigments continuously in the light and darkness, but reached a much higher cell density in the light (Figure 5A). This finding is in clear contrast to results obtained with laboratory cultures of several representative aerobic photosynthetic alphaproteobacteria, which accumulated BChl *a* exclusively in the dark [7]. Consequently, generalizing statements postulating a stringent inhibition of BChl synthesis in marine aerobic anoxygenic photosynthetic bacteria even by low light intensities [*e.g.* 36], [37] have to be reconsidered. Interestingly, our data are in line with early results of Kolber et al. [38] who found only a minor effect of light on pigment synthesis in aerobic bacteriochlorophyll-containing bacteria in marine ecosystems, but a diel growth cycle in oxygenic phototrophs with chlorophyll synthesis restricted to periods of darkness. In contrast to growth on L-malate pigment synthesis of KT71^T^ cells was hardly detectable during growth in the complex medium SYPG. It appears that trace amounts of pigments were only synthesized during the stationary phase thereby inducing a minimal increase of cell density (Figure 5B). Thus, the data presented indicate a light-dependent stimulation of growth due to photosynthesis. Further evidence for this assumption was obtained by growing pigmented and unpigmented KT71^T^ cells under identical conditions in defined semiaerobic medium containing 6 mM DL-malate as substrate (Table 1).

**Table 1:** Inhibition of KT71^T^ cultures growing on different substrates by the addition of fluoroacetate.

Medium/Substrate	A_660 nm_	A_880 nm_/A_660 nm_	Inhibition of Growth with		
			0.4 mM FAc	2.0 mM FAc	4.0 mM FAc
SMP	0.196	0.62	100%	100%	100%
2 mM Sucrose	0.188	0.54	95%	100%	99%
6 mM Pyruvate	0.134	0.55	96%	100%	100%
6 mM Oxaloacetate	0.138	0.55	90%	100%	n.d.
6 mM DL-Malate (pigmented phenotype)	0.381	0.92	26%	59%	61%
SYPG	0.329	0.60	25%	33%	43%
4 mM Pentanoate	0.087	0.54	0%	19%	56%
6 mM DL-Malate (unpigmented phenotype)	0.150	0.54	9%	8%	6%

Fluoroacetate was added to cultures at the beginning of the exponentially growth phase. All cultures were incubated at 28°C with an initial oxygen concentration of 12 vol% under dim light. The growth inhibition caused by a distinct amount of fluoroacetate compared to growth in normal medium was calculated by using the following formula: Inhibition (%) = [1−(A_660_ t_s_−A_660_ t_0_)FAc/(A_660_ t_s_−A_660_ t_0_)]×100, where A_660_ is absorption at 660 nm, t_s_ the time at which the culture without fluoroacetate reached the stationary phase, t_0_ the time at which fluoroacetate was added and FAc designates the culture supplemented with fluoroacetate. n.d., not determined.

In Table 2 the percentage of substrate assimilation upon growth with various single carbon sources is compared for different strains. The percentage of carbon assimilation obtained by KT71^T^ in the dark with acetate or L-malate as substrate was unusually low, at least compared to *Silicibacter pomeroyi* and *R. rubrum*. The low growth yield could either indicate an incomplete utilization of these substrates or an uncoupling of substrate oxidation and energy generation. This observation could provide a first clue on the function of photosynthesis in this strain, if we assume that additional energy generated from light is required for the efficient utilization of certain substrates with a low energy yield. A light-dependent increase in growth yield in cultures of KT71^T^ was only observed if a photosynthetic apparatus was expressed, but there was no arithmetic correlation of the growth yield increase with the abundance of the photosynthetic apparatus, so that some additional factors may have influenced the efficiency of photophosphorylation. Based on the dry weight values obtained growth yield estimates could be made for KT71^T^ and compared with data obtained with *Silicibacter pomeroyi* or *R. rubrum*. It was found that in KT71^T^ the molar growth yield on malate (*Y_malate_*) was 18.6 g (dw) in the dark and 44.2 g (dw) in the light. Assuming that the amount of biomass formed per mol ATP is constant for a certain strain cultivated in the same medium [39] it can be deduced that under optimal conditions KT71^T^ cells incubated in the light produce around 2.4 times more ATP than cultures incubated in the dark. With malate as substrate the growth yield of KT71^T^ cultures in the light was considerably higher than in the non-photosynthetic species *Silicibacter pomeroyi*, but smaller than in *R. rubrum* cultures grown anaerobically in the light. Nevertheless, aerobic photosynthesis still increased the growth yield of KT71^T^ cultures growing on malate by a factor of approximately 1.4 compared to *Silicibacter pomeroyi*, which is close to the estimate of Kolber et al. [1] for the light-induced growth yield increase of aerobic anoxygenic photosynthetic bacteria in the upper open ocean.

**Table 2:** Carbon assimilation by strain KT71^T^.

Strain	Incubation	Percentage of Substrate Carbon Assimilation			
		Acetate	L - Malate	Succinate	D - Galactose
KT71^T^	Aerobic, Dark	18 (0.55)	19 (0.89)	22 (0.85)	37 (0.60)
KT71^T^	Aerobic, Light	18 (0.53)	46 (0.93)	28 (0.91)	62 (0.62)
S-1^T^ a	Aerobic, Dark	46	37	37	n.r.
S-1^T^ a	Anaerobic, Light	90	69	82	n.r.
DSM 15171^T^	Aerobic, Dark	33	32	42	no growth

The proportion of assimilated carbon in KT71^T^ upon growth on different substrates is compared with the phototrophic purple non-sulfur bacterium *Rhodospirillum rubrum* S-1^T^ ( = DSM 467^T^) and the chemoheterotrophic alphaproteobacterium *Silicibacter pomeroyi* DSM 15171^T^. Batch cultures of KT71^T^ and DSM 15171^T^ were incubated with 2 mM L-malate, 2 mM succinate, 1 mM D-galactose and 3 mM acetate under an initial oxygen concentration of 6 vol% at 28°C. KT71^T^ and DSM 15171^T^ were adaptated to various carbon sources by one transfer from SMFC medium in defined medium containing the respective carbon source prior to inoculation of the main culture. Numbers in parentheses denote A_880 nm_/A_660 nm_ values determined in KT71^T^ cultures at early stationary phase. n.r., not reported.

a Data for *R. rubrum* are from Clayton [88].

### Parameters Affecting Photoheterotrophic Growth

#### Carbon source utilization

**Figure 6:**
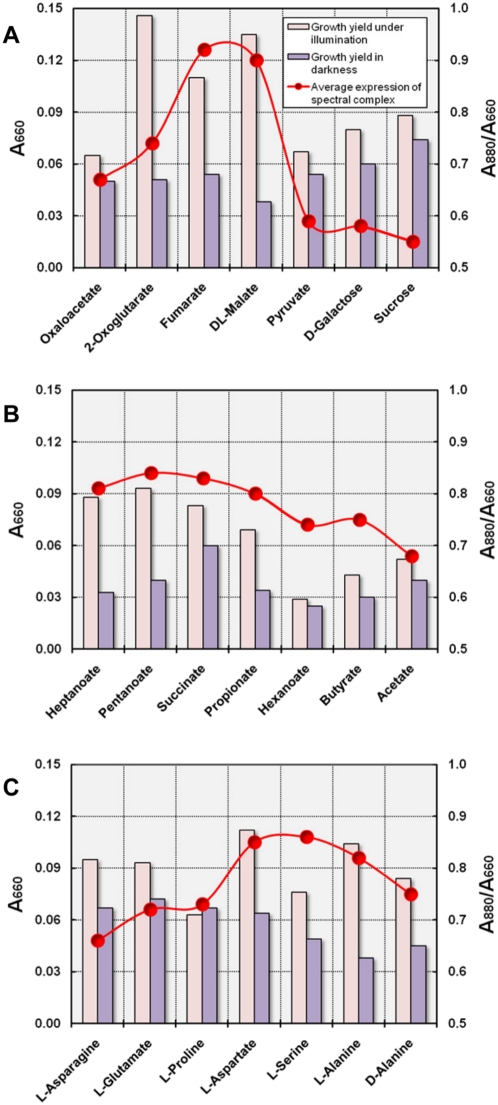
Dependency of the expression of the photosynthetic apparatus on carbon source utilization. The effect of the utilization of sugars and citric acid cycle intermediates (A), fatty acids (B) and amino acids (C) on pigment production is shown. All cultures were incubated in defined medium containing 2 mM of the respective carbon source at 6 vol% initial oxygen concentration at 28°C. The growth yield was determined spectrophotometrically (A_660 nm_) in the stationary phase following incubation in dim light or darkness. Expression of the spectral complex in stationary phase grown cells was estimated by determination of A_880 nm_/A_660 nm_ values. The circles shown represent average values of two cultures incubated with the same carbon source, one incubated in light and the other in darkness.

Most species of aerobic anoxygenic photosynthetic bacteria are routinely cultured in only one type of medium, so that the effect of carbon source utilization on the expression of the photosynthetic apparatus is largely unknown. In addition, often media were used that contain complex nutrients like yeast extract or peptones, so that the substrate utilization pattern cannot be exactly determined. Strain KT71^T^ did not express significant amounts of photosynthetic pigments during subcultivation in complex SYPG medium, but expressed a photosynthetic apparatus if malate was used as sole carbon source. In some experiments an induction of pigment synthesis was also observed after transfer from SYPG medium to defined media containing other carbon sources like succinate or oxoglutarate, but not with oxaloacetate or pyruvate. A detailed analysis of the induction of pigment synthesis in KT71^T^ was however hampered by several adversities. For instance, the transfer of cultures to defined media containing single carbon sources did not always lead to the induction of pigment synthesis and caused normally a prolonged lag phase, so that results could not be easily reproduced. Hence, we decided to study the effect of carbon source utilization with pigmented cells that were pre-grown under photoheterotrophic conditions. Using this approach a change of pigment expression occurred promptly and could be reliably determined. It was found that the level of pigment expression in cultures grown under dim light in SMFC medium at an initial oxygen tension of 6 vol% was quite stable and ranged between 0.85 and 0.88 (A_880 nm_/A_660 nm_ values in stationary phase), so that preparatory cultures were routinely obtained by subcultivation in this medium. In a series of experiments more than twenty different substrates were identified that can be utilized by KT71^T^ as a single carbon source and resulted in different levels of pigment expression. In Figure 6 the cell density and pigmentation of stationary phase cultures is shown following transfer from SMFC medium in defined medium containing 2 mM of carbon source. KT71^T^ is able to utilize a wide range of substrates including citric acid cycle intermediates and sugars (Figure 6A), fatty acids (Figure 6B) and amino acids (Figure 6C). Overall, the obtained data indicate that the expression of photosynthetic pigments in defined medium depends mainly on the position at which substrates are funneled into the citric acid cycle. Most substrates that enter the central metabolism at the starting point of the citric acid cycle, *i.e.* as acetate or oxaloacetate, caused a severe repression of pigment production, which resulted in unpigmented cells after several transfers in the same medium. In contrast, fumarate or malate that both are formed at the end of this cycle stimulated the expression of a photosynthetic apparatus. The amino acids L-serine and L-alanine caused no significant repression of pigment synthesis (Figure 6C), although they were likely degraded to pyruvate, which acted as a strong inhibitor of photosynthesis. The reason could be an unexpected pathway for the assimilation of these amino acids or a more complex interaction of pigment expression and carbon metabolism in KT71^T^.

#### Concentrations of substrate and oxygen

**Figure 7:**
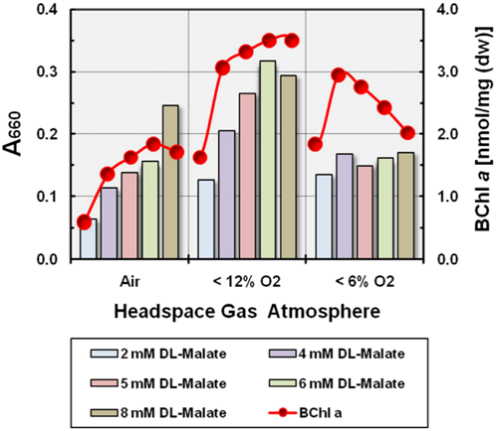
Effect of substrate and oxygen concentration on growth yield and production of photosynthetic pigments. Cultures were incubated at 28°C in dim light under fully aerobic, semiaerobic and microaerobic conditions. Substrate concentrations ranged from 2 to 8 mM DL-malate. The amount of produced bacteriochlorophyll *a* was determined spectrophotometrically in acetone/methanol extracts from wet cell pellets obtained by harvesting cultures grown to stationary phase.

It was originally thought that members of the marine OM60/NOR5 clade represent obligately oligotrophic bacteria unable to grow in media with high nutrient concentrations [17], [40]. However, we have found that strain KT71^T^ can be gradually adapted to growth in liquid or solid media containing more than 5 g l^−1^ of complex substrates. Although, an increase in the length of the lag phase was observed upon transfer to media with very high nutrient concentrations this effect was probably not due to oligotrophic metabolism, but instead may indicate a slow adaptation of the gene expression pattern to changing growth conditions. An extended lag phase was also observed for example, if cultures were transferred to media containing different carbon sources in low concentrations. In addition, more than twenty phylogenetically diverse isolates affiliated to this group were isolated recently from Wadden Sea sediment off Sylt, Germany (unpublished results), which grew on isolation very well in SYPG medium and to a higher density as in the oligotrophic medium described by Eilers et al. [12]. Hence, the results obtained with strain KT71^T^ are presumably not based on a genetic drift caused by repeated subcultivation in the laboratory. However, independent of the general growth response there could be still a possible effect of substrate concentration on the expression level of photosynthesis genes as assumed by Kolber et al. [1] and Cho et al. [17]. To test this hypothesis we used batch cultures of KT71^T^ that were incubated under fully aerobic, semiaerobic and microaerobic conditions with various amounts of malate as sole carbon source. Photosynthetic pigments were extracted from cultures grown to stationary phase and quantified by spectrophotometry. First we tested, if an increase of the carbon source concentration has a negative effect on the growth yield. The results are shown in Figure 7 and indicate no negative effect on the growth yield by an increase of the malate concentration provided the oxygen concentration is not a growth limiting factor. At an initial oxygen concentration of 6 vol% the growth yield with all tested concentrations of malate was similar, but merely because the availability of oxygen limits growth. This becomes clear from the observation that the growth yield increased proportional with the malate concentration under an air atmosphere. In Figure 7 it is shown that the amount of BChl *a* produced increased with the substrate concentration under semiaerobic growth conditions and reached maximal levels with 6 or 8 mM malate. Thus, in the presence of an oxygen surplus the expression level of photosynthetic pigments increased with the amount of substrate utilized, which is in clear contradiction to the proposed stimulation of aerobic anoxygenic photosynthesis by nutrient limitation. In general, it appears that the growth yield and pigment production depended on the initial malate/oxygen ratio and were optimal under semiaerobic conditions, provided that neither the carbon nor oxygen concentration is a growth limiting factor. In contrast to our finding Cho et al. [17] reported in their study of strain HTCC2080, which is a member of the OM60/NOR5 clade and closely related to KT71^T^ (Figure 1), that light-dependent growth stimulation occurred only in medium with very low concentrations of organic substrates. However, it has to be noted that the substrate composition in their oligotrophic medium (based on autoclaved sea water containing 80 µM dissolved organic carbon) was most likely not identical to the artificial mixture of substrates that was used for the preparation of carbon-rich media containing 3.0 or 14.7 mM dissolved organic carbon, so that the effect on photosynthesis may well have been caused by a different substrate utilization pattern.

Conditions of severe carbon or oxygen limitation led to a reduction in growth yield and inhibition of pigment production in KT71^T^. This effect may be explained by a correlation of the membrane redox state with the efficiency of energy generation. At an optimal carbon to oxygen ratio the membrane redox carriers are likely to be in a balanced state, which could promote the establishment of an active Q-cycle. In contrast, under conditions of severe oxygen limitation respiration is inhibited and redox carriers will be predominantly in a reduced state, whereas the combination of a high oxygen concentration with a very low carbon concentration will give rise to a potential overoxidation of the electron transport chain. Thus, the observed growth yield optimum under semiaerobic conditions is in agreement with the assumed involvement of a Q-cycle in photophosphorylation, which requires a balanced membrane redox state for optimal efficiency [41]. On the other hand, it can be deduced that KT71^T^ has only a limited ability to balance the cellular redox state actively in order to optimize the membrane redox state for the generation of energy.

#### Light intensity and quality

A major environmental parameter that controls the expression of photosynthesis genes in anoxygenic photosynthetic bacteria is light. In most studies it was found that illumination with dim light (below 1000 lux) stimulates expression of a light-harvesting apparatus, whereas high light-intensities (above 10,000 lux) cause a repression of pigment synthesis [42], [43]. The reason behind this type of regulation is probably to increase the amount of absorbed photons under non saturating conditions for photosynthesis and on the other hand to avoid photooxidative damage by strong light in the presence of oxygen. In some species of aerobic anoxygenic photosynthetic bacteria affiliated to the *Alphaproteobacteria* an almost complete repression of photosynthetic pigments even at intermediate light intensities of 4000 lux [44] or 20 µE m^−2^ s^−1^ [7] was observed. In contrast, some preliminary results obtained with members of the OM60/NOR5 clade indicated that expression of pigments is repressed in darkness and stimulated by light [10], [17]. To find out if light is indeed a requirement for the expression of pigments in KT71^T^ we incubated various defined media in complete darkness after inoculation with cells grown photoheterotrophically in SMFC medium. To determine the stability of pigment expression cultures were transferred to fresh medium for at least five times in the dark. The results obtained are illustrated in Figure S2 and indicate that after several transfers in darkness the level of pigment expression decreased in all tested media. While the expression of pigments was stable in cultures of KT71^T^ growing with malate in the light, continuous incubation in the dark resulted in the almost complete loss of photosynthetic pigmentation (A_880 nm_/A_660 nm_ value of 0.57) after six successive transfers. The unpigmented phenotype was stable even during prolonged incubation in the light and hence could be used for a comparison with photoheterotrophically growing cells in a series of experiments described below. In Figure S2 it is also shown that the significance of this effect depended largely on the carbon source utilized. In cultures growing on succinate or fumarate a high amount of photosynthetic pigments was still produced after several transfers in darkness, whereas with propionate the synthesis of pigments was completely repressed after three successive transfers, with oxaloacetate or pyruvate as carbon source already upon the first transfer. Interestingly, cultures growing with 2-oxoglutarate showed a variable repression of pigments during incubation in darkness, which indicates that the regulation of pigment synthesis is unstable under these conditions and depends not only on illumination. Hence, it can be concluded that light is not a prerequisite for pigment expression, but stimulates it indirectly, perhaps by interaction with the photosynthetic electron transport system as suggested by Happ et al. [45].

**Figure 8:**
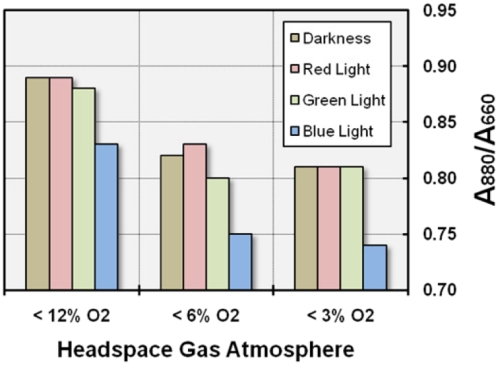
Effect of illumination and light quality on the expression level of the photosynthetic apparatus. Cultures were grown in SMFC medium and incubated at 23°C in complete darkness or in a dark cabinet under illumination with LED bulbs emitting monochromatic red, green or blue light. Average values of two independent experiments are shown.

On the other hand, it could be shown in several preliminary experiments that light has a negative effect on pigment expression, provided the intensity is above 10,000 lux. Illumination of cultures growing in SMFC medium under semiaerobic conditions with high-intensity white light (11,000 lux, equivalent to 154 µE m^−2^ s^−1^) emitted from fluorescent lamps (Osram T8 L 58W/865 Lumilux Daylight) led to strong inhibition of pigmentation or even prevented growth (data not shown). Consequently, the effect of light on the level of photosynthetic pigment expression in KT71^T^ is not fundamentally different from the situation in most facultative anaerobic anoxygenic phototrophs. To determine the impact of light on the synthesis of pigments in more detail, cultures growing in SMFC medium were incubated in darkness or under illumination with various LED spotlights emitting either red, green or blue light with peak wavelengths of 627, 518 and 466 nm, respectively. An effective illuminance of 5500 lux was measured, if the green LED lamp was used. Based on the results summarized in Figure 8 it can be deduced that only blue light had a significant effect on pigment expression, which was independent of the initial oxygen concentration. Consequently, it is likely that any possible sensors that are involved in the light-dependent regulation of pigment synthesis in KT71^T^ sense blue light.

#### Citric acid cycle activity and intermediary carbon metabolism

It can be assumed that the correlation of pigment expression and carbon metabolism in KT71^T^ is a complex interaction, because two different stable phenotypes growing with malate as sole carbon source could be obtained in the course of this study. The unpigmented phenotype growing on malate was obtained from photoheterotrophically growing cultures by repeated subcultivation in the dark (Figure S2). There is a possibility that the unpigmented phenotype is based on a loss of photosynthesis genes, but this is not probable, because an expression of photosynthesis genes in unpigmented cells could be demonstrated by reverse transcription-PCR of mRNA, albeit at a lower level (unpublished results). To gain more information about the possible role of the intermediary carbon metabolism for the expression of photosynthesis genes in KT71^T^, we tried to analyze the citric acid cycle activity in cells growing on various substrates by using the specific inhibitor fluoroacetate. After entering the cell fluoroacetate is converted to fluoroacetyl-CoA by an acetyl-CoA synthetase (annotated ORF: KT71_08034). This compound is used by citrate synthase instead of acetyl-CoA to form fluorocitrate, which then inhibits specifically the enzyme aconitase thereby leading to an interruption of the citric acid cycle [46]–[48]. Fluoroacetate was used previously in several studies to eliminate citric acid cycle activity in resting cells and to determine the fraction of reduction equivalents that can be produced independently of this pathway [*e.g.* 49]. In our experiments fluoroacetate was added to cultures that already entered the exponential growth phase, so that a substrate specific intracellular carbon balance was established before fluoroacetate entered the cells. The response of KT71^T^ to the addition of fluoroacetate was tested using eight different medium compositions. The data obtained are summarized in Table 1. In Figure S3 representative growth curves are shown, which were obtained by incubation of KT71^T^ in four different media supplemented with varying amounts of fluoroacetate. Growth inhibition caused by fluoroacetate did not correlate directly with the level of pigment expression observed in parallel cultures incubated without fluoroacetate. Interestingly, the effect of fluoroacetate on cells of the unpigmented phenotype growing with malate as carbon source was markedly different to the effect caused on pigmented cells growing in the same medium, which probably indicates a different pattern of substrate utilization. Supposing that the amount of intracellulary synthesized fluorocitrate is independent of the incubation conditions, the data obtained would imply that generation of NAD(P)H by the citric acid cycle depends mainly on the utilized substrate and is almost absent in cells growing chemoheterotrophically with malate, pentanoate or in SYPG medium. On the other hand, this pathway would be indispensable for cells growing chemoheterotrophically in SMP medium or in defined media containing sucrose, pyruvate or oxaloacetate. Furthermore, growth in the absence of significant citric acid cycle activity would indicate that these cells do not have a respiratory metabolism, which depends on the production of NADH by the oxidative citric acid cycle. However, this appears unlikely, because it was shown that KT71^T^ is an obligately aerobic microorganism and expresses a respiratory electron transport chain with terminal oxidases under all tested incubation conditions. In addition, growth behavior in various deep agar cultures indicated an aerobic metabolism with all tested substrates (Figure S4). Based on these results we conclude that the metabolism is always respiratory and thus requires an active citric acid cycle for the production of the required amount of reduction equivalents. Hence, there must be another reason for the dependence of fluoroacetate inhibition on substrate utilization. We propose the following explanation: Kelly [48] has shown that fluoroacetate-resistant cells of *Halothiobacillus neapolitanus* (formerly *Thiobacillus neapolitanus*) are characterized by a lack of acetyl-CoA synthetase activity. Furthermore, he demonstrated that an excess of extracellular acetate prevents inhibition by fluoroacetate, but only if it is present in the medium prior to addition of fluoroacetate. This would mean that acetate has to be metabolized before fluoroacetate enters the cell in order to prevent growth inhibition. We conclude from these observations and our data, that substrates that lead to a depletion of the intracellular coenzyme A pool and an increase of acetyl-CoA or other coenzyme A thioesters induce a resistance against fluoroacetate by repression of the acetyl-CoA synthetase activity, which is necessary for the formation of fluoroacetyl-CoA. On the other hand, substrates that lead to an increase of the intracellular coenzyme A to acetyl-CoA ratio would stimulate acetyl-CoA synthetase activity and fluoroacetate toxicity. This theory can explain why KT71^T^ cells growing with pentanoate are rather resistant to fluoroacetate, whereas cells growing with oxaloacetate are highly sensitive. Pentanoate is metabolized to acetyl-CoA and propionyl-CoA thereby leading to a depletion of the coenzyme A pool, whereas an excess of oxaloacetate would lead to a decrease of the acetyl-CoA concentration by the citrate synthase reaction. Based on this assumption, we can conclude that the high fluoroacetate sensitivity of cells growing on pyruvate or sucrose is probably due to an intracellular accumulation of oxaloacetate. Oxaloacetate can be synthesized from pyruvate or phosphoenolpyruvate, which are intermediates of glycolysis, by carboxylation reactions. The key enzyme for this pathway in KT71^T^ is phosphoenolpyruvate (PEP) carboxylase (annotated ORF: KT71_12695), which forms oxaloacetate and phosphate by the carboxylation of PEP. It is known that this anaplerotic reaction represents a major route for CO_2_ assimilation in heterotrophic and mixotrophic bacteria [6], [9]. In addition, it can be deduced that the repression of pigment synthesis in cultures growing with malate in the dark correlates with an increase of the acetyl-CoA concentration, so that the intracellular activation of fluoroacetate is prevented. A rise of the acetyl-CoA concentration in KT71^T^ cells growing with malate could be caused for example by an induction of malic enzyme (annotated ORF: KT71_12670), which decarboxylates malate to pyruvate that can be subsequently converted to acetyl-CoA by the pyruvate dehydrogenase complex. On the other hand, it is likely that the presence of pyruvate in SMP medium leads to a feedback inhibition of malic enzyme and stimulation of pyruvate orthophosphate dikinase (annotated ORF: KT71_12340) and PEP carboxylase, thereby leading to the accumulation of oxaloacetate and depletion of acetyl-CoA.

Based on these considerations, we propose that the level of pigment expression in KT71^T^ depends partly on the intracellular carbon metabolism and is maximal under conditions of a balanced carbon flux, that is when neither acetyl-CoA nor oxaloacetate are accumulating.

### Identification of Cellular Redox Monitors

Based on the results presented above it is possible that the expression of photosynthesis genes in KT71^T^ depends mainly on the cellular redox state. Therefore, it would be useful to estimate the redox state in growing cells and to correlate these values with the expression of the photosynthetic apparatus. However, the reliable determination of the redox state in bacterial cells is difficult and technical demanding. Alternatively, it should be possible to identify distinct phenotypic traits, in addition to the expression of photosynthetic pigments, that allow an estimation of the cellular redox state. It is known, for example, that in facultative anaerobic bacteria several metabolic pathways are under control of redox sensing proteins, like FNR or OxyR, which could play also a role in the expression of phenotypic traits in KT71^T^. In general, the cellular redox state of aerobically growing cells depends on two antagonistic effects: The substrate dependent production of metabolic reductants (mainly reduced pyridine nucleotides) decreases the cellular redox state, whereas ambient oxygen, which is required to oxidize NADH in aerobic respiration, penetrates the cell membrane and leads to an increase of the intracellular redox state. Hence, we decided to use the following criteria for the identification of phenotypic traits as potential redox monitors: On the one hand, these phenotypic traits should vary with the oxygen tension, but then should depend also on the type of substrate or amount of substrate utilized at a constant oxygen tension. Based on these criteria several putative redox monitors could be identified, which are described below.

#### Oxygen demand of growing cells

Previously, it was found that the oxygen concentration within a layer of cells that formed in deep agar cultures of KT71^T^ is around 30 µmol O_2_ l^−1^ and quite stable, although its position relative to the air/agar interface varied and correlated with the amount of substrate utilized [10]. Hence, it is likely that the position of the visible band of bacteria in the agar medium reflected the oxygen demand of growing cells. Cells which require a high amount of oxygen to balance their redox state will be situated closer to the agar surface than cells requiring only a small amount of oxygen. Based on these considerations, we expected that inoculation of semisolid agar media containing various substrates will result in different patterns of band formation depending on the potential of substrates to stimulate oxygen consumption.

In Figure S4 the positions of formed bands in deep agar cultures containing various substrates is shown. In cultures growing in SYPG or SMP medium or with sucrose or pyruvate as substrates the position of the cell layer was close to the agar surface, whereas with acetate, oxaloacetate or malate (unpigmented phenotype) it was located several millimeters below the surface, thereby indicating a lower oxygen demand. The position of the band that formed in cultures with cells growing photoheterotrophically with malate as substrate was located at an intermediate position. Hence, we conclude that unpigmented cells growing on acetate, malate or oxaloacetate have a lower respiratory activity than cells growing with sucrose or pyruvate as substrate.

#### Cytochrome c oxidase activity

**Figure 9:**
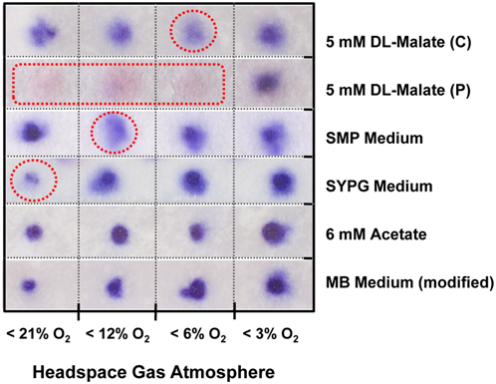
Estimation of the cytochrome *c* oxidase activity in whole cells upon growth in various media. Strips of filter paper containing blotted cells are shown after soaking with TMPD solution. The intensity of the blue stain correlates with the cytochrome *c* oxidase activity. Spots indicating minimal activity are enclosed by a red circle. C, chemoheterotrophically grown unpigmented cells; P, photoheterotrophically grown cells.

The cytochrome *c* oxidase activity in bacteria can be roughly quantified by the amount of N,N,N′,N′-tetramethyl-*p*-phenylenediamine (TMPD) that becomes oxidized to a blue colored derivative immediately upon contact with whole cells. It is assumed that this reaction is specific for cells expressing an active cytochrome *c* oxidase, because strains lacking such an enzyme usually show only a weak reaction upon exposure to TMPD, which can be used as electron donor instead of cytochrome *c* by terminal oxidases [50]. We have found that the oxidase activity in strain KT71^T^ was highly variable and changed with the initial oxygen tension as well as with the substrate utilized (Figure 9), which indicates a correlation with the cellular redox state. An identification as redox monitor was however complicated by the fact that the amount of oxidized TMPD correlated not proportionally with the oxygen concentration, but in most cases displayed a minimum at semiaerobic growth conditions. In Figure 9 it is shown that the oxygen concentration at which a minimal cytochrome *c* oxidase activity was found depended on the substrate utilized and was relatively high (<21 vol% O_2_), if cells were grown in the complex media MB or SYPG and rather low (<6 vol% O_2_), if they were grown chemoheterotrophically in defined medium containing malate as sole carbon source. We deduced from this observation that the oxidase activity reaches minimal values under conditions of a balanced redox state and increases as the redox state becomes more reduced or oxidized. Thus, at oxygen-limiting conditions (6 vol% initial oxygen concentration) high oxidase activities should indicate either an overreduced or overoxidized cellular redox state. It can be deduced from Figure 9 that cells growing with acetate are probably in an overreduced state, which is unexpected because the amount of reduction equivalents that can be derived from this carbon source should be lower than from most other substrates. It is also noteworthy, that the oxidase activity in photoheterotrophically growing cells remained almost completely repressed under various initial oxygen concentrations ranging from 6 to 21 vol%, which could indicate that the redox state is actively kept in a balanced state.

#### Composition of the electron transport chain

**Figure 10:**
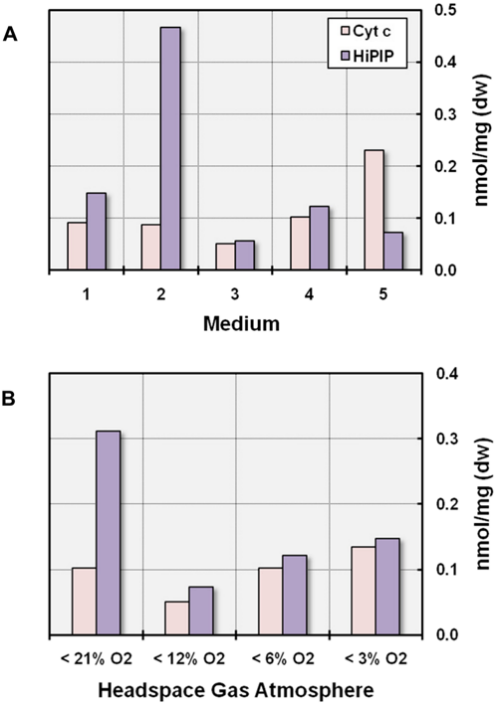
Variable utilization of cytochromes *c* and putative high-potential iron-sulfur proteins as electron donors in respiration. The amount of cyt *c* and HiPIP participating in respiratory electron transfer was deduced from cyanide-treated *minus* air-oxidized difference spectra. (A) Effect of photosynthesis and substrate utilization on the composition of the electron transport chain. All cultures were incubated at 28°C under an initial oxygen concentration of 6 vol%. (1) medium with 5 mM DL-malate (unpigmented cells, darkness); (2) medium with 5 mM DL-malate (pigmented cells, dim light); (3) SMP medium (unpigmented cells, darkness); (4) SYPG medium (unpigmented cells, darkness); (5) modified MB medium (unpigmented cells, darkness). (B) Effect of the oxygen concentration on the respiratory electron transport chain. All cultures were incubated in SYPG medium in darkness.

The observed variation of the cytochrome *c* oxidase activity in KT71^T^ cells should be reflected in a variable biochemical composition of the electron transport chain. Hence, we analyzed the diversity of electron transport chains in this strain by redox difference spectroscopy of whole cells or extracts treated with various inhibitors that interrupt the electron transfer at different sites. In Figure S1 results are shown that were obtained either with cells grown photoheterotrophically on malate or with unpigmented cells that were grown chemoheterotrophically in SMP or SYPG medium or with malate as substrate. Potassium cyanide was used to inhibit terminal oxidases (Figure S1B). Addition of cyanide to solubilized cell extracts should result in the reduction of the inhibited oxidase and its primary electron donor [51]. It could be shown that in solubilized extracts of cells grown in SYPG medium about 90% of the total high-potential cyt *c* could be reduced by the addition of cyanide. On the other hand, in photoheterotrophically grown cells only a small fraction of solubilized cyt *c* was reducible by the addition of cyanide and a characteristic featureless bleaching of the spectrum in the region between 450 and 550 nm was observed. This feature is typical for reduced iron-sulfur proteins [52] and could indicate that under conditions of photoheterotrophic growth cyt *c* is replaced by an alternative periplasmic redox carrier, likely a high-potential iron-sulfur protein (HiPIP). In a complementary experiment stigmatellin was used to inhibit reduction of cyt *c* by the ubiquinol-cytochrome *c*-oxidoreductase (Figure S1C). A significant oxidation of cyt *c* was only found in difference spectra (dithionite and stigmatellin *minus* dithionite) of chemoheterotrophically growing unpigmented cells. In cells expressing a photosynthetic apparatus the spectrum was dominated by redox active photosynthetic pigments, whereas signals indicating presence of *c*-type cytochromes were hardly detectable. To analyze the expression of terminal oxidases carbon monoxide (CO) was added to whole cell suspensions (Figure S1D). CO can be used for the detection of oxidases because it binds specifically to heme moieties that have oxygen binding capacity. However, the identification of distinct oxidases in whole cells is difficult and usually restricted to cells expressing only one type of oxidase. Nevertheless, a pronounced variability in difference spectra (dithionite and CO *minus* dithionite) obtained with pigmented and unpigmented cells was evident. Apparently, signals derived from CO-binding *c*-type cytochromes (trough at 550–553 nm) were most intense in photosynthetic cells and less pronounced in unpigmented cells grown in SMP and SYPG medium or with malate as substrate. In chemoheterotrophically grown cells a visible trough at 443–447 nm in the Soret region indicated expression of an oxidase containing heme *a*. However, an additional characteristic trough at around 610 nm caused by the ferrocytochrome *a_3_*-CO complex was not detected. The presence of terminal oxidases containing heme *b* was deduced from a negative shoulder around 564 nm and a trough at 428 nm in the Soret region of the difference spectrum, which could be most clearly detected in photoheterotrophically grown cells. Based on the obtained results, we conclude that the intensity of the oxidase reaction correlates with signals indicating presence of an active *aa_3_*-type oxidase in redox difference spectra. Hence, it is possible that during photoheterotrophic growth or under conditions of a balanced redox state expression of the *aa_3_*-type cytochrome *c* oxidase is inhibited, whereas a *cb*-type oxidase characterized by a low TMPD turnover rate is stimulated. In Figure 10 the estimated amounts of cytochromes *c* and high-potential iron-sulfur proteins participating in the respiratory electron flow are shown and can be correlated with the results of the TMPD assay. It appears that growth conditions that are characterized by a participation of HiPIPs in the respiratory chain correlate with a low TMPD oxidase activity. These data would indicate that HiPIPs in KT71^T^ are not only likely candidates for the reduction of the reaction center cytochrome *c* (especially due to the lack of alternative donors like cyt *c*_2_ or *c*_8_), but may also play a role as electron donors for a *cb*-type terminal oxidase under conditions of a balanced redox state or during photoheterotrophic growth.

We hypothesize that this *cb*-type oxidase could represent a novel HiPIP∶oxygen oxidoreductase, which cannot utilize TMPD as electron donor. The existence of a HiPIP∶oxygen oxidoreductase in betaproteobacteria was also previously proposed by Hochkoeppler et al. [53], who found that respiration in the facultative phototroph *Rhodoferax fermentans* depends on the oxidation of HiPIP by a *cb*-type terminal oxidase. Alternatively, a low TMPD oxidase activity of whole cells could be caused by the expression of ubiquinol oxidases. However, no genes encoding potential ubiquinol oxidases could be identified in the KT71^T^ draft genome sequence.

#### Cellular fatty acid composition

**Figure 11:**
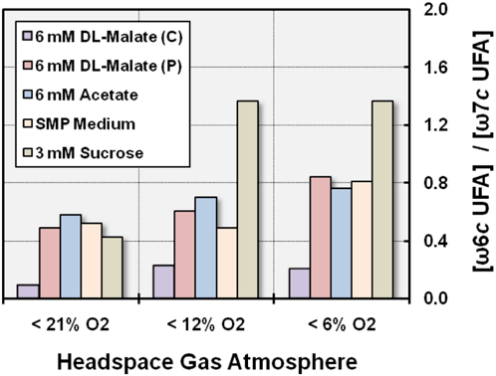
Variable ratios of ω6*c* to ω7*c* monounsaturated cellular fatty acids in response to different redox regimes. Cultures were incubated with various substrates at 28°C for 5–7 days under illumination with dim light. C, chemoheterotrophically grown unpigmented cells; P, photoheterotrophically grown cells; UFA, unsaturated fatty acid.

A detailed analysis of the cellular fatty acid composition under various incubation conditions revealed a significant dependence on substrate utilization and the availability of oxygen. This indicates that the production of distinct cellular fatty acids could reflect the cellular redox state. In Table S2 it is shown that the position of the double bond in the prevalent unsaturated fatty acids changed from ω6 (counted from the methyl end) at microaerobic conditions to ω7 under conditions close to oxygen saturation, which could indicate two different pathways for the synthesis of unsaturated fatty acids that are controlled by the cellular redox state. Accordingly, under the same oxygen tension the amount of synthesized ω6 fatty acids depended on the medium used and reached higher levels upon incubation with the nutrient-rich MB medium than with malate as carbon source (Table S2). In Figure 11 the ratios of ω6*c* to ω7*c* unsaturated fatty acids in cells that were grown under various incubation conditions are shown. The data presented could indicate that cells growing with sucrose as carbon source have a lower redox state than cells growing in SMP medium or with acetate and that photoheterotrophically growing cells have a more reduced redox state than unpigmented cells growing chemoheterotrophically in the same medium. In addition, one may assume from the data obtained that the overall redox state in unpigmented cells growing with acetate or in SMP medium under semiaerobic conditions is comparable to that in photoheterotrophically growing cells. This would indicate that the expression of photosynthesis genes in strain KT71^T^ is not only controlled by the overall redox state, but depends also on the composition or activity of the electron transport chain, which has been shown to be different in photosynthetically active and unpigmented cells.

#### Pigment stoichiometry

**Figure 12:**
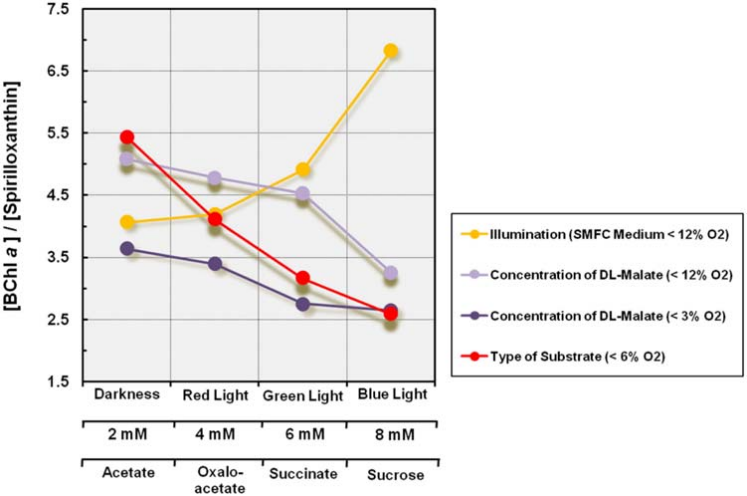
Molar ratio of bacteriochlorophyll *a* (BChl *a*) to spirilloxanthin in cells grown under various incubation conditions. Unless indicated otherwise cultures were illuminated with dim white light from an incandescent bulb. Average values obtained in two independent experiments are shown in the graph illustrating the influence of light quality on the pigment composition.

It is known that the redox state of the electron transport chain is reflected by the reduction of the quinone pool, which in turn is influenced by the type of substrate, availability of oxygen and light intensity in photoheterotrophic bacteria [54]. It has been shown, for instance, that oxygen depletion or an increase of the substrate concentration led to an increased reduction of the quinone pool in *Escherichia coli* [55] and *R. rubrum* [56]. In Figure 12 the pigment composition of photoheterotrophically growing KT71^T^ cells is correlated with a variation of growth conditions suggested to alter the reduction of the cellular ubiquinone pool. The data presented can be interpreted in such a way that conditions, which lead to an increased reduction of the quinone pool lead to a decrease of the BChl *a* to spirilloxanthin ratio. Consequently, transfer of photoheterotrophically grown cells to medium containing acetate as substrate resulted in a more oxidized quinone pool than transfer to a medium with sucrose. As both substrates induced a drastic reduction of the pigment production, it was not possible to correlate the repression of photosynthesis genes with a distinct change of the BChl *a* to spirilloxanthin ratio. Thus, one might assume that the reduction of the quinone pool represents a signal that controls mainly the pigment stoichiometry, but has only a minor influence on the level of pigmentation.

Interestingly, we have also found that illumination has a strong influence on the pigment stoichiometry. An analysis of the pigment composition in cells grown on various carbon sources revealed that upon growth in dim light the molar ratio of BChl *a* to spirilloxanthin was on average 62% higher than upon incubation in complete darkness, while the expression level of the photosynthetic apparatus (deduced from A_880 nm_/A_660 nm_ values) remained almost constant. Although, the values determined varied considerably the tendency of this effect was quite stable and could be reproduced in numerous independent experiments. As shown in Figure 12 this effect was not restricted to an illumination with blue light, but increased with decreasing wavelength of the light source. Hence, it appears that the pigment stoichiometry in KT71^T^ is influenced by signals that depend on the reduction of the quinone pool instead of being controlled by a light-sensing protein. Klamt et al. [54] have proposed that the quinone pool in photosynthetically active proteobacteria is more reduced in darkness than under illumination, which would be in line with our findings.

A possible explanation for the redox-sensitive regulation of the pigment stoichiometry in strain KT71^T^ could be as follows: As shown above this strain does not express a peripheral light-harvesting (LH2) complex, which could be used to adapt the absorption of photons to light intensity as observed for example in *Rhodobacter* species that produce variable amounts of the LH2 complex in response to changing light intensities [57], [58]. Instead, strain KT71^T^ may control the amount of photons absorbed not only by the abundance of the LH1 complex, but also by the spirilloxanthin content of the light-harvesting apparatus. Some meters below the water surface in coastal areas the majority of the penetrating photons is in the range between 400 and 550 nm [9] and thus absorbed by carotenoids and not bacteriochlorophyll *a*, so that a reduction of the carotenoid content could help to avoid photooxidative damage by illumination under conditions of a high redox state. This finding is unexpected, because it has been a frequently repeated assumption that aerobic anoxygenic photosynthetic bacteria accumulate large amounts of carotenoids in order to prevent photooxidative damage caused by reactive oxygen species. However, a large part of the carotenoids produced in these species is not involved in light harvesting and only loosely associated with the photosynthetic apparatus [59]. Hence, the strategy of KT71^T^ may be more economical under conditions of energy deprivation and nutrient limitation.

## Discussion

### Correlation of Photosynthesis with the Cellular Redox State

In facultatively anaerobic photosynthetic proteobacteria several redox balancing mechanisms are present that prevent a decrease of the cellular redox state to unfavorable levels. These systems are either based on enzymes of the electron transport chain like ubiquinol oxidase and fumarate reductase or metabolic pathways like the assimilation of CO_2_ by the Calvin-cycle and fixation of nitrogen that require a large amount of reduction equivalents and ATP [60]. In KT71^T^ none of these mechanisms could be detected, neither by annotation of the draft genome sequence nor by physiological tests, so that no redox balancing system except for the universal thioredoxin buffer system [61] seems to be present. The identification of cellular redox monitors in KT71^T^ allowed us to estimate the cellular redox state of KT71^T^ cells growing on various substrates. It turned out that the intracellular redox state in this strain is variable and depends largely on the growth conditions. In the course of this study it was not possible to detect a principal correlation of the substrate type with an assumed cellular redox state, which would be based merely on theoretical considerations like a different utilization of biochemical pathways. However, most of the obtained results can be explained intuitively in the following way: We assume that the redox state in KT71^T^ is mainly controlled by the amount of reduction equivalents derived from the substrate and the respiratory activity. The utilization of highly reduced substrates like sucrose induces in cells an intracellular accumulation of NADH along with a high respiratory activity, whereas the utilization of substrates with a very low energy yield like acetate causes an inhibition of the respiration rate in order to avoid a harmful overoxidation of the cellular redox state, which in turn leads also to an intracellular accumulation of reduction equivalents. In some cases, if substrates with an intermediate energy yield like malate are utilized the redox state may drop to suboptimal levels, which, however, can still be tolerated without causing an inhibition of respiration. Most of the obtained data indicate that one of the main reasons for the repression of photosynthetic pigment production in KT71^T^ is a significant decrease or increase of the cellular redox state compared to the redox state in photoheterotrophically growing cells. Candela et al. [41] have demonstrated that the generation of ATP in photosynthetic membranes requires a balanced redox state that corresponds to a reduction of the membrane-bound quinone pool of about 50%, which is optimally for the promotion of a photosynthetic Q-cycle. In addition, they have shown that an overreduction or overoxidation of the membrane redox state leads not only to a prevention of the light-induced generation of energy in facultative anaerobic, but also in aerobic photosynthetic proteobacteria. Hence, the expression of photosynthesis genes in bacteria representing both types of metabolism only makes sense, if it is possible to establish a balanced membrane redox state for an extended period of time. This finding could provide an explanation for the assumed correlation of photosynthesis gene expression with the cellular redox state in KT71^T^. Further support for this hypothesis was obtained from the observation that treatment of cultures with chemicals that prevent the oxidation of NADH (KCN) or induce oxidative stress (paraquat) resulted both in a strong repression of pigment production in KT71^T^ cells (unpublished results). It follows that any sensor-regulator protein that controls the expression of photosynthesis genes in this strain should be able to sense equally well an overreduction and overoxidation of the cellular redox state. We propose that the PpsR protein (annotated ORF: KT71_19353) is the most likely candidate to fulfill this function. The PpsR protein encoded in the genome of KT71^T^ contains two adjacent PAS (Per-Arnt-Sim) domains, a C-terminal HTH (helix-turn-helix) motif probably mediating DNA binding and two cysteine residues at positions 349 and 412, which are thought to play a key role in redox sensing along with the PAS domains [62]. DNA binding sites of PpsR can be recognized by the conserved palindromic sequence TGTcaN_8_tgACA [63]. In several species studied two adjacent palindromes are found overlapping with putative σ^70^ – type promoters where the binding of PpsR causes a repression of photosynthesis genes located downstream of the operator sequences [63]. An analysis of the PGS nucleotide sequence of KT71^T^ revealed a total of 15 putative PpsR binding sites, of which 12 were arranged in a typical tandem orientation separated by seven or eight nucleotides (Figure S5). The number of binding sites is comparable to that found in facultative anaerobic phototrophs [64] and indicates that PpsR plays a pivotal role in controlling the transcription level of photosynthesis genes in KT71^T^. The following observations reported previously about the PpsR protein in other purple bacteria seem to support the view that PpsR can operate as “bidirectional” redox sensor: In *Rhodobacter sphaeroides* the PpsR protein was found to bind DNA not only in the oxidized state, but also under reducing conditions [65], which is in clear contrast to its proposed function as an aerobic repressor [62], but in line with our model of a protein sensing a distinct redox state. In addition, the PpsR1 protein in the strictly aerobic *Bradyrhizobium* strain ORS278 was found to have a higher DNA affinity in its reduced than in its oxidized form [66]. Hence, one intrinsic property of the PpsR protein architecture seems to be the ability to bind DNA under reducing and oxidizing conditions. It is known that the redox balancing system of facultative anaerobic purple bacteria prevents an overreduction of the cellular redox state under anaerobic conditions in the light, so that normally only the aerobic repression of photosynthesis genes is observed in laboratory cultures. Consequently, it could well be that the repression of photosynthesis genes due to an overreduction of the redox state was not realized in most studied photosynthetic proteobacteria and thus not studied in detail so far.

In addition to the PpsR protein *Rhodobacter* species contain a two-component response regulator system (RegA/RegB or PrrA/PrrB), which is not present in KT71^T^. In *Rhodobacter sphaeroides* the two-component activation system PrrBA is controlled by a *cbb_3_*-type terminal oxidase [67]. Increasing concentrations of oxygen enhance the electron flow through the *cbb_3_* oxidase, which generates a signal that prevents phosphorylation of the PrrA protein by the membrane-bound PrrB histidine kinase. This leads to a repression of photosynthesis, because the phosphorylated form of PrrA is required to stimulate transcription of photosynthesis genes [68]. We have found that in KT71^T^ the expression of a photosynthetic apparatus coincided with the expression of an active *cbb_3_*-type terminal oxidase. We speculate that this oxidase senses the electron flow under conditions of a balanced redox state and generates a signal that stimulates expression of photosynthesis genes. A potential signal could be the intracellular concentration of the second messenger c-di-GMP, because in the genome of KT71^T^ a large gene encoding GGDEF/EAL output domains (annotated ORF: KT71_16971) was found upstream close to the operon encoding a *cbb_3_* oxidase of the sensing type (annotated ORFs: KT71_16991 – KT71_17006). It is known that proteins containing GGDEF/EAL domains control the level of c-di-GMP in bacterial cells [69]. However, the mechanisms that control the activity of these proteins are still largely unknown and could for example involve an interaction with terminal oxidases.

In the draft genome of KT71^T^ two genes were annotated that encode a BLUF (blue-light-using flavin adenine dinucleotide) domain [70], [71], which could have a function in the light-dependent control of pigment expression. One of both genes (annotated ORF: KT71_19323) is located close to the gene encoding PpsR. The negative effect of blue light on the expression level of the photosynthetic apparatus was quite independent from the initial oxygen tension, which indicates that probably no additional redox sensor is involved in the light-dependent regulation of pigment expression as it was shown in *Rhodobacter sphaeroides*. In this species repression of pigment synthesis in response to blue light depends on the interaction of the AppA protein that simultaneously senses oxygen and light with the PpsR repressor [45], [72]. However, no open reading frame with significant similarity to the *appA* gene was detected in the draft genome sequence of KT71^T^, so that this type of light-dependent regulation of pigment expression is not applicable to this bacterium. A protein with a single BLUF domain was also identified in the genome of the aerobic anoxygenic photosynthetic bacterium *Roseobacter denitrificans* in which pigment synthesis was repressed selectively by illumination with blue light [7], although no gene with similarity to the *appA* gene was identified [64]. Hence, it may be speculated that in some photosynthetic proteobacteria the single domain BLUF protein could interact with PpsR in an unknown way in order to control pigment synthesis in response to the light intensity.

In conclusion, it appears that the PpsR protein represents a terminal effector of a complex regulatory network that depends on the cellular redox state, activity of terminal oxidases, quinone reduction and light intensity. Consequently, the induction of photosynthesis genes would only occur if optimal conditions are sensed by various signal cascades, which could prevent a frequent and energy expensive change of the gene expression pattern. A summary of the proposed model for the regulation of photosynthesis in KT71^T^ is presented below:

1. KT71^T^ represents a heterotrophic, obligately aerobic photosynthetic bacterium lacking a redox balancing system, which results in a variable cellular redox state that depends mainly on the type of utilized substrate, intermediary carbon metabolism and oxygen concentration.
2. Some unidentified cellular signals that correlate with the redox state and/or the respiratory activity are sensed by the transcription regulator protein PpsR, which has the function to repress the transcription of photosynthesis genes under conditions that lead to an overreduced or overoxidized membrane redox state, because both states prevent an efficient generation of energy from light.
3. A single domain BLUF protein acts as a sensor of blue light and modulates the activity of the PpsR protein in a yet unknown way.
4. The pigment stoichiometry in photosynthetically active cells is controlled by the reduction of the ubiquinone pool, which depends mainly on the availability of oxygen and light.
5. Photosynthesis in KT71^T^ may function to prevent an unfavorable overoxidation of the cellular redox state by photoreduction of NAD^+^ involving an energy dependent reverse electron flow.

In the future more advanced analyses of the gene expression in KT71^T^ are planned to verify the postulated metabolic model and to arrive eventually at a complete understanding of the molecular mechanisms that control the expression of photosynthesis genes in members of the abundant OM60/NOR5 clade within the *Gammaproteobacteria*.

### Taxonomic Status of Strain KT71^T^

In reconstructed phylogenetic trees strain KT71^T^ belongs to a branch comprising the recently described species *Haliea salexigens* and *Spongiibacter marinus* as well as numerous 16S rRNA gene sequences retrieved from uncultured marine bacteria (Figure 1). An affiliation of this sequence cluster to any family described currently within the class *Gammaproteobacteria* is not supported by high bootstrap values in reconstructed maximum-parsimony or distance-matrix trees. Thus, members of this group represent most likely a novel family. Similarity values between the 16S rRNA gene sequences of KT71^T^ and the most closely related species *Haliea salexigens* and *Spongiibacter marinus* are only 95.4 and 91.0%, respectively, thereby excluding a relationship at species level [73]. In addition, the presented data clearly indicate that strain KT71^T^ can be distinguished phenotypically from related genera, so that we propose the formal classification in a novel genus and species. A comparison of distinguishing features of *Haliea salexigens* and the proposed species *Congregibacter litoralis* is shown in Table 3 and formal descriptions are presented below.

**Table 3:** Distinguishing phenotypic traits of *Congregibacter litoralis* KT71^T^ and *Haliea salexigens* 3X/A02/235^T^.

Characteristic	KT71^T^	3X/A02/235^T^
Isolation source	North Sea (8 m water depth)	Mediterranean Sea (water surface)
Cell shape	pleomorphic	straight rods
Cell size (µm)	0.5–4.5×0.4–0.7	1.3–1.9×0.3–0.7
Flagellation type	1–2 (sub)polar	1 polar
**Growth Characteristics**		
NaCl range (%)	1–7	0.7–7
NaCl optimum (%)	2	4.2
Temp. range (°C)	9–33	10–37
Temp. optimum (°C)	28	25–30
pH range	6.5–9.0	5.0–9.0
pH optimum	7.5–8.0	8.0
Anaerobic Growth	−	−
Growth on marine agar 2216	+	+
Photosynthetic pigments	+	n.d.
Oxidase	+	+
Catalase	(+)	+
**Carbon Source Utilization**		
Cellobiose	−	−
Galactose	+	−
Glucose	−	−
Sucrose	+	−
Glycerol	+	+
Acetate	(+)	−
Citrate	−	−
3-Hydroxybutyrate	+	+
Propionate	(+)	−
Pyruvate	+	+
Succinate	+	+
Alanine	+	−
Aspartate	+	+
Glutamate	+	(+)
Proline	+	+
Serine	+	−
**Chemotaxonomy**		
Major fatty acids	16∶0, 18∶1, 16∶1, (17∶1)	17∶1, 16∶1, 18∶1
Major 3-OH fatty acid	10∶0 3OH	11∶0 3OH
Polar lipids	PE, PG, PL	DPG, PG, APL
Quinone	UQ8	UQ8
G+C content of DNA (mol%)	57.8	61.4

Data for *H. salexigens* were taken from Urios et al. [13]. +, positive; (+), weakly positive; −, negative; n.d., not detected; PE, phosphatidylethanolamine; PG, phosphatidylglycerol; PL; unknown phospholipid; DPG, diphosphatidylglycerol; APL, unknown aminophospholipid.

### Description of *Congregibacter* gen. nov

*Congregibacter* (Con.gre.gi.bac'ter. L. adj. *congregus -a -um*, united in flocks; N.L. masc. n. *bacter*, a rod; N.L. masc. n. *Congregibacter* a rod that grows in flocks).

Cells are Gram-negative, non-spore-forming and multiply by binary fission. Aggregates are frequently formed in liquid medium under suboptimal growth conditions, especially carbon starvation [10]. Mesophilic and moderately halophilic. Strictly aerobic, respiratory and heterotrophic metabolism. Tests for oxidase and catalase activity are positive. Cytochromes of the *c*-type are dominating in redox difference spectra. Bacteriochlorophyll *a* and carotenoids of the spirilloxanthin series are produced in photosynthetically active cells. In the presence of photosynthetic pigments light stimulates growth under semiaerobic conditions. The production of photosynthetic pigments is not repressed in aerobically growing cells by illumination with dim light, *i.e.* below 2000 lux of incandescent light (equivalent to 40 µE m^−2^ s^−1^). Under certain incubation conditions water-insoluble polar pigments with a pale yellow to orange-red color can be formed. Major cellular fatty acids are C_16∶0_, C_16∶1_ and C_18∶1_. The dominating hydroxy fatty acid is C_10∶0_ 3OH. Ubiquinone 8 represents the sole respiratory lipoquinone. Phosphatidylethanolamine, phosphatidylglycerol and an unidentified phospholipid are the major polar lipids. Representatives can be found in sea water and the surface layer of littoral marine sediments.

The type species is *Congregibacter litoralis*.

### Description of *Congregibacter litoralis* sp. nov

*Congregibacter litoralis* (li'to.ra.lis. M.L. adj. *litoralis* from the shore, pertaining to the habitat from where the organism was isolated).

In addition to traits noted for the genus the following characteristics were determined. Cells are pleomorphic and depending on the growth conditions either coccoid or irregular rod-shaped with rounded ends. The length of cells can vary between 0.5 and 4.5 µm and the width between 0.4 and 0.7 µm. Motility is conferred by one or two polar to subpolar flagella. The reserve polymers cyanophycin [10] and polyphosphate are stored in intracellular granules. The color of culture suspensions is variable and reflects mainly the production of photosynthetic pigments. Under conditions that allow maximal expression of BChl *a* and spirilloxanthin suspensions are pale-pink to deep-red depending on the cell density. Yellow-orange or cream-white suspensions are obtained, if the expression of pigments is repressed. In fully pigmented photoheterotrophically growing cells a rudimentary intracytoplasmic membrane system of the lamellar type is formed. Living cells of photoheterotrophically growing cultures showed absorption maxima in the near-infrared region of the spectrum at 802 and 876 nm indicating the presence of a reaction center and light-harvesting 1 complex. Colonies appear on plates of marine agar (DIFCO 2216) after 48 to 72 hours incubation at 28°C and can reach a size of 1–2 mm. They have a round shape with regular edges and are cream colored, thin, slightly convex and soft. Growth was observed between 9 and 33°C and at sea salts concentrations ranging from 1 to 15% (w/v). In media containing 10 mM MgSO_4_ the range of suitable NaCl concentrations was 1–7% (w/v) with an optimum at 2% (w/v) NaCl. The requirement for salts is complex and sodium, chloride and either magnesium or calcium ions were needed for growth. No growth occurs below pH 6.0 or above pH 9.5. Optimal conditions for growth are 28°C, a sea salts concentration of around 4% (w/v) and a pH value between 7.5 and 8.0. The mean generation time under optimal growth conditions is 4.5 h. Facultatively microaerophilic; high oxygen concentrations inhibit growth under oligotrophic conditions. No growth under anaerobic conditions with fumarate, nitrate, ferric iron, sulfur, thiosulfate or sulfate as terminal electron acceptors or by fermentation. Excretion of a transparent slime, which causes an increase of medium viscosity, was frequently observed upon incubation in liquid nutrient-rich complex media under conditions of oxygen saturation. The polymers casein, gelatin, starch, cellulose, alginate, agar and DNA were not degraded. Growth depends on organic substrates that are utilized as carbon and energy source; no growth was observed with hydrogen, thiosulfate, sulfur or ferrous iron as electron donor and CO_2_ as carbon source. The following compounds were used for growth: *alkanes:* decane (weak), dodecane (weak) and octane (weak); *alcohols:* glycerol and propanol (weak); *carboxylic acids:* acetate (weak), butyrate (weak), dodecanoate, heptanoate, DL-3-hydroxybutyrate, hexanoate (weak), DL-malate, oleate, oxaloacetate, 2-oxoglutarate, palmitate, pentanoate, pyruvate, propionate (weak) and succinate; *amino acids:* D-alanine, L-alanine, D-arginine (weak), L-arginine, L-asparagine, D-aspartate (weak), L-aspartate, L-cysteine (weak), L-glutamate, glutathione, L-proline and L-serine; *carbohydrates:* D-galactose and sucrose. The following compounds were tested, but not utilized: *alkanes:* hexane, hexadecane and tetradecane; *alcohols:* meso-erythritol, ethanol, myo-inositol, D-mannitol, methanol and resorcinol; *carboxylic acids:* acrylate, 2-aminobenzoate, benzoate, citrate, decanoate, formate, glycolate, DL-lactate and octanoate; *amino acids:* L-cysteate, DL-glycine, L-histidine, L-isoleucine, L-lysine, L-methionine, L-ornithine, L-phenylalanine, D-proline, D-serine, L-valine and taurine; *carbohydrates:* L-arabinose, cellobiose, D-fructose, D-glucose and melibiose. Tests were positive for tweenase and urease activity, but negative for tryptophanase, arginine dihydrolase and esculinase activity. Degradation of L-cysteine or glutathione did not result in the release of sulfide. Suitable nitrogen sources for growth were ammonium, urea, amino acids and purines, but not molecular nitrogen, nitrate, taurine or thiourea. Requirement for the vitamins thiamine, biotin and B-12. Sensitive to the antibiotics cefalotin, imipenem, chloramphenicol, gentamicin, neomycin, colistin and polymyxin B; resistant to oxacillin, tetracycline, doxycycline, vancomycin, lincomycin and bacitracin. The DNA G+C content of the type strain is 57.8 mol% (HPLC).

The type strain is KT71^T^ ( = DSM 17192^T^ = NBRC 104960^T^). It was isolated from the water column (8 m depth) of the North Sea near Helgoland (Germany).

## Materials and Methods

### Strains used, media and incubation conditions

The strains *Congregibacter litoralis* KT71^T^ ( = DSM 17192^T^), *Rhodospirillum rubrum* DSM 467^T^, and *Silicibacter pomeroyi* DSM 15171^T^ were used in this study. Pure chemicals were obtained from Sigma-Aldrich (http://www.sigmaaldrich.com) and complex nutrients from DIFCO BBL (http://www.vgdusa.com/DIFCO.htm). Unless otherwise noted the subcultivation of DSM 467^T^ and DSM 15171^T^ was done using the media and incubation conditions given in the DSMZ (http://www.dsmz.de) catalogue of strains. The authenticity of strain KT71^T^ was controlled in regular intervals by determining MALDI-TOF spectra of whole cell protein extracts as described by Cousin et al. [74].

Subcultivation of strain KT71^T^ was routinely carried out in media based on the following mineral solution: 35.0 g sea salts (Sigma) and 1.0 ml Wolfe's mineral elixir (see DSMZ medium 792) dissolved in 1000.0 ml demineralized water. The mineral salt solution was prepared under an atmosphere of air or under 80% N_2_ and 20% CO_2_ gas atmosphere using the anaerobe culture technique of Hungate [75] with the modifications introduced by Bryant [76]. To avoid precipitation of salts in the mineral solution prepared under an atmosphere of air the pH was adjusted to 5.5 using 1 M HCl prior to autoclaving. Substrate stock solutions were prepared anoxically under 100% N_2_ gas atmosphere and sterilized by filtration. The mineral salt solution was dispensed in 120 ml (total volume) serum vials, closed with oxygen tight butyl rubber stoppers and sealed with aluminum crimps prior to autoclaving. After autoclaving 0.10 g l^−1^ NH_4_Cl, 0.05 g l^−1^ KH_2_PO_4_, 10.00 ml l^−1^ vitamins solution (see DSMZ medium 141) and various substrates were added aseptically to the mineral solution from anoxic stock solutions. After completion of the medium the pH was adjusted to 7.4–7.8 using Na_2_CO_3_ added from a sterile anoxic stock solution (5% w/v) prepared under 80% N_2_ and 20% CO_2_ gas atmosphere.

The mineral salt solution was supplemented with various carbon sources to yield the following media used for routine subcultivation of KT71^T^: The complex medium SYPG contained yeast extract (0.50 g l^−1^), trypticase peptone (0.25 g l^−1^) and sodium L-glutamate (0.10 g l^−1^) as substrates. Copiotrophic conditions were achieved by adding 5.0 g l^−1^ peptone and 1.0 g l^−1^ yeast extract to the mineral salt solution which is equivalent to the amounts used in DIFO marine broth (MB) medium 2216. The defined medium SMP contained as substrates 3 mM of disodium DL-malate (0.53 g l^−1^) and 3 mM of sodium pyruvate (0.33 g l^−1^). Photoheterotrophic growth in the light was achieved by using the defined SMFC medium that contained 4 mM disodium DL-malate (0.71 g l^−1^) and 100 µM ferric citrate (0.034 g l^−1^). Cultures were incubated under various headspace gas atmospheres containing 3 to 21 vol% oxygen at the start of growth. Conditions close to oxygen saturation were achieved by filling 30 ml air-saturated medium in 120 ml serum vials that were sealed under air atmosphere. Alternatively, 250 ml Erlenmeyer flasks were filled with 50 ml aerobic medium and closed using foam stoppers to allow free exchange with the air atmosphere. To obtain headspace gas atmospheres that correspond approximately to an initial oxygen concentration of 12 or 6 vol%, serum vials were filled under air atmosphere with 52 or 83 ml anoxic medium, respectively. For a concentration of 3 vol% oxygen 22 ml of sterile air was injected into closed anoxic serum vials containing 41 ml of medium. After an equilibration period of several hours the resulting overpressure was released from the vials. Assuming an oxygen density of 1.33 g l^−1^ (20°C, 1 atm.) the absolute amount of oxygen per vial was calculated to be approx. 5.2, 10.3, 19.0, and 24.1 mg at an initial headspace concentration of 3, 6, 12, and 21 vol%, respectively. Due to the different volumes of medium within the vials the weight ratios of substrate to oxygen were almost identical at 3 and 6 vol% O_2_, but approx. three times less compared to vials with 12 vol% O_2_ and six times less compared to vials with 21 vol% initial oxygen concentration. No attempt was made to determine the effective concentration of dissolved oxygen in the liquid medium. Cultures incubated in serum vials were shaken by hand at least every second day, whereas Erlenmeyer flasks were agitated continuously at 100 rpm on a rotary shaker. In cultures incubated in sealed vials growth is accompanied by a continuous decrease in oxygen concentration, which is indicated in the lettering of figures and tables by a “less than” symbol (<) in front of the initial oxygen concentration. In contrast, cultures growing in Erlenmeyer flasks or on agar plates were in equilibrium with the air atmosphere and the difference to an incubation in sealed vials was made clear by using the word “air” instead of 21 vol% oxygen. For reasons of simplicity in the discussion of experiments initial head space gas atmospheres of 3–6 vol% oxygen were designated as microaerobic, 12–21 vol% O_2_ as semiaerobic, and incubation under air atmosphere as fully aerobic.

The correlation of oxygen consumption and substrate utilization in KT71^T^ was estimated by cultivation in soft agar tubes containing resorufin as redox indicator. The medium for the deep agar cultivation was prepared as described by Fuchs et al. [10]. A similar approach was used by Leadbetter and Breznak [77] to test oxygen tolerance of *Methanobrevibacter* species. They could show by oxygen microsensor measurements that decolorization of resorufin indicates oxygen free conditions within the agar gradients.

The percentage of carbon assimilation upon growth on various substrates was determined in batch cultures that were incubated in custom made 1200 ml serum bottles filled with 830 ml of anoxic medium under air atmosphere resulting in an initial oxygen concentration of approx. 6 vol%. The calculated amount of oxygen per serum bottle was approx. 103 mg or 3.2 mmol O_2_. In batch cultures a molar carbon (C) to molecular oxygen (O_2_) ratio of 1.5–2 at the start of growth was found optimal for substrate assimilation by KT71^T^ at an initial oxygen concentration of 6 vol%. Hence, substrate amounts that comply with 4.5 to 6.5 mmol organic carbon were selected for the carbon assimilation experiments.

Unless indicated otherwise cultures were illuminated with dim white light from an Osram (http://www.osram.com) 40 W tungstate incandescent bulb resulting in a light intensity of about 1400 lux (equivalent to 28 µE m^−2^ s^−1^). Spectra of LED spotlights (Osram Decospot) emitting red (0.67 W), green (0.82 W) or blue light (0.84 W) were kindly determined by Craig Johnson (http://www.ledmuseum.org) using a USB2000 spectrometer with SpectraSuite software (http://www.two-cubed.com). The luminous emittance of the light sources used was determined with a Gossen (http://www.gossen-photo.de/) Lunasix 3 light meter.

### Measurement of growth yield and expression of the spectral complex

The absorbance values of growing cultures were determined in a LKB Biochrom Ultrospec II 4050 UV/visible spectrophotometer using 1 cm light path disposable cuvettes and water as blank. The A_660 nm_ reading was used to estimate the cell density in KT71^T^ cultures. Culture density of *R. rubrum* was measured also at 680 nm to obtain values that can be compared with literature data. The A_880 nm_ to A_660 nm_ ratio was used to determine the abundance of the LH1 complex in living cells. According to Grammel et al. [78] A_880 nm_/A_660 nm_ values of 1.2 correspond with suspensions of fully pigmented cells of *R. rubrum*, whereas values below 0.58 are mainly due to the wave length dependent scattering of light and hence indicate suspensions of cells lacking a LH1 complex.

For the determination of *in vivo* spectra of cell suspensions of KT71^T^ or *R. rubrum* sucrose was added to a final concentration of 0.65 g ml^−1^ to early stationary phase cultures adjusted to an A_660 nm_ of approx. 0.2. Whole cells absorption-spectra were recorded with a Perkin Elmer (http://www.perkinelmer.com) Lambda 2 split beam spectrophotometer.

The cellular dry weight of grown cultures was determined by overnight freeze-drying of cell pellets harvested by centrifugation. A comparison of the determined cellular dry weights with corresponding absorbance values revealed a constant ratio of 0.70 mg dry weight per absorbance unit at 660 nm for strain KT71^T^ cultured in defined media containing various carbon sources (R^2^ = 0.992, n = 6). Upon growth in the complex medium SYPG a somewhat smaller ratio of 0.53 mg dry weight/A_660 nm_ was obtained (R^2^ = 0.993, n = 4). Based on empirical data reported in the literature it was assumed that carbon accounts for 50% of the cellular dry weight.

### Physiological and morphological characterization

General physiological tests were determined according to the protocols described by Gerhardt et al. [79]. The utilization of substrates was tested at 28°C in defined medium containing 2 mM of the respective compound as sole carbon source. Media prepared were inoculated with 1% (v/v) of a KT71^T^ culture grown to stationary phase in SMFC medium. For each substrate parallel cultures were incubated under an initial oxygen concentration of 6 vol% in dim white light (1400 lux) and darkness. After an incubation period of 10–30 days OD values (A_660 nm_) obtained in the two cultures were determined. The following criteria were applied to estimate the growth response: An average OD_660_ value above 0.05 was usually obtained upon good growth on the tested substrate, whereas a value between 0.03 and 0.05 indicated only weak growth and values below 0.02 indicated no significant growth. In every case substrates that supported growth were utilized both in the light and in darkness. In defined media without substrate no growth was observed.

For ultrastructural analysis cells of strain KT71^T^ were either grown chemoheterotrophically or photoheterotrophically and treated for embedment in ERL-resin [80]. Energy-filtered transmission electron microscopy (EF-TEM) analysis at zero-loss was done with post-stained 90 nm [81] and untreated 35 nm ultrathin sections either in the elastic bright-field (0 eV) or 250 eV high-contrast imaging (HCI) mode as described in detail by Lünsdorf et al. [82] with a Zeiss CEM 902 at 80 kV.

### Determination of photosynthetic pigments

Cells were harvested by centrifugation and the supernatant carefully removed from the cell pellet. After determination of the wet weight cells were frozen at −20°C. Pigments were extracted from the wet cell pellet with 700 µl acetone/methanol (7∶2) for 4 hours at 5°C under nitrogen atmosphere in the dark. The extract was either completely filled in 1 cm light path quartz cuvettes or diluted prior to measurement to obtain maximal absorbance values at 771 nm below 0.5. Spectra were recorded with a Perkin Elmer Lambda 2 split beam spectrophotometer.

The concentrations of bacteriopheophytin *a*, bacteriochlorophyll *a* and spirilloxanthin in the acetone/methanol extracts were determined from the absorbance values obtained at 747, 771 and 475 nm, respectively. The spectral reconstruction method of van der Rest and Gingras [26] was used to determine pigment concentrations in the extracts obtained applying extinction coefficients for bacteriopheophytin *a*, bacteriochlorophyll *a* and spirilloxanthin of 45.1 mM^−1^×cm^−1^, 65.3 mM^−1^×cm^−1^ and 94.0 mM^−1^×cm^−1^, respectively.

### Analysis of the membrane composition

Cellular fatty acid patterns were determined from cells grown to stationary phase in defined medium containing various substrates or in DIFCO 2216 marine broth. Cultures were incubated under various gas atmospheres with initial oxygen concentrations ranging from 6 to 21 vol%. The preparation and extraction of fatty acid methyl esters from biomass and their subsequent separation and identification by gas chromatography was done as described by Kaksonen et al. [83]. Lipoquinones and polar lipids were extracted and separated according to the methods described by Tindall [84] using a Shimadzu HPLC apparatus (http://www.shimadzu.com/) fitted with a reverse phase C18 column (150 mm×4.6 mm (ID), 5 µm, porosity 90 Å, Vydac) with UV detection at 269 nm.

### Identification and quantification of redox active proteins

The dominant cytochrome types were identified in photoheterotrophically (pigmented) and chemoheterotrophically grown (unpigmented) KT71^T^ cells by determining difference spectra with a Perkin Elmer Lambda 2 split beam spectrophotometer. First, cells were harvested in early stationary phase from a culture volume that equals a biomass of approx. 7 mg dry weight (corresponding to an A_660 nm_×volume (ml) of 10.0 or 13.3 depending on the growth medium) and suspended in 1.5 ml Tris/NaCl buffer (3.2 g l^−1^ TRIS-HCl, 21.0 g l^−1^ NaCl, 2.5 g l^−1^ MgCl_2_×6H_2_O, 1.0 g l^−1^ KCl, 0.2 g l^−1^ CaCl_2_×2 H_2_O [pH 7.8]). For the determination of the cytochrome types in KT71^T^ cells grown in SYPG medium under various oxygen concentrations a culture volume equaling an A_660 nm_ of 6.0 was used. Cells were lysed and membranes solubilized by adding 0.3% w/v of the non-ionic detergent N,N-dimethyldodecylamine-N-oxide (LDAO). The resulting suspension was incubated at room temperature for approx. 15 min. until complete lysis occurred. The crude extract obtained was then cleared from large cell debris by a centrifugation step (14,000 rpm for 5 min.). The resulting supernatant was then used to obtain redox difference spectra. First, the suspension was oxidized with 0.1 mM K_3_Fe(CN)_6_, distributed in two 0.70 ml, 1 cm light path, quartz cuvettes and the baseline determined. Thereafter, one cuvette was reduced with 10 mM ascorbate and measured against the oxidized cuvette until the spectrum was stable. Then, 1–2 mg of sodium dithionite were added to the cuvette containing ascorbate and again measured against the oxidized cuvette. Pyridine hemochrome spectra were obtained by dissolving cell pellets (approx. 7 mg dw) in a 3∶1 mixture (v/v) of 0.2 M NaOH and pyridine. According to Bartsch [85] pellets of pigmented cells were extracted several times with acetone/methanol (7∶2 v/v) prior to dissolving in pyridine-NaOH solution to remove pigments. A rough estimate of the cytochrome *c* and cytochrome *a* concentrations in sample cuvettes containing solubilized membranes was achieved by using the difference extinction coefficients Δε_552-538_ = 17.3 mM^−1^×cm^−1^ [32] and Δε_604-630_ = 25.2 mM^−1^×cm^−1^ [86], respectively. To quantify the concentration of pyridine hemochrome *a* the difference extinction coefficient Δε_588-620 nm_ = 25.0 mM^−1^×cm^−1^ was used [87]. The abundance of putative high-potential iron-sulfur proteins was estimated using the difference extinction at 480 nm (∼9 mM^−1^×cm^−1^ [52]).

The effect of cyanide on the activity of terminal oxidases was analyzed by adding potassium cyanide to solubilized cell extracts. Culture samples equivalent to an A_660 nm_×volume (ml) of 12.0 were harvested and the cell pellet resuspended in 1.5 ml Tris-NaCl buffer. Cells were lysed by adding LDAO as described above. The suspension was distributed in two cuvettes and a baseline representing air-oxidized samples was determined. Then, 1 mM KCN was added to the sample cuvette and a difference spectrum to the native air-oxidized sample determined. After an incubation period of 5–10 min. a stable spectrum was obtained and the total amount of high-potential cytochromes *c* was determined by adding 10 mM ascorbate to the sample cuvette.

For the detection of an ubiquinol-cytochrome *c* oxidoreductase activity the specific inhibitor stigmatellin was used. A stock solution of stigmatellin was prepared in absolute ethanol. Solubilized cell extracts were prepared as described above, reduced with 1–2 mg sodium dithionite and distributed in two cuvettes. A small amount of inhibitor (about 100 µmol) was added to a sample cuvette and the same amount of ethanol to the reference cuvette. After an incubation period of 5–10 min. a difference spectrum was obtained.

Binding of carbon monoxide to terminal oxidases was determined in whole cell suspensions. Cells from a culture volume that equals an A_660 nm_ of 6.0 were harvested by centrifugation and suspended in 0.75 ml Tris/NaCl buffer. Thereafter, sucrose was added to a final concentration of 0.65 g ml^−1^ to minimize light scattering. After sucrose has been completely dissolved, the suspension was reduced with 1–2 mg of sodium dithionite, distributed in two cuvettes and the baseline determined. Subsequently, one cuvette was gently bubbled with 100% CO gas for approx. 5 min. and then, following an incubation time of 10 min., measured against the reference cuvette reduced only with dithionite. The cytochrome c oxidase activity of whole cells was determined by oxidation of N,N,N′,N′-tetramethyl-*p*-phenylenediamine (TMPD) dihydrochloride which results in the formation of a blue stain. Cells equivalent to an A_660 nm_×volume (ml) of 0.25 were harvested by centrifugation and spotted on a piece of filter paper (Schleicher & Schuell, Nr. 2294). The underside of the paper strip was then soaked with a 0.5% (w/v) solution of TMPD in water. After an incubation period of 30–60 sec. a photograph was taken to document development of a blue stain.

A search for related proteins in various public domain databases was done using the BLAST tool provided by the *National Center for Biotechnology Information* site at http://www.ncbi.nlm.nih.gov/blast/Blast.cgi.

## Supporting Information

Table S1 Relevant genes encoding proteins of the KT71^T^ electron transport chain. ORF, open reading frame; MW, molecular weight in Dalton; pI, isoelectric point. ^a^ Designation of the putatively expressed protein with the proposed gene abbreviation in parentheses. ^b^ Designation of functional sites is based on domain annotation given by INTERPRO (http://www.ebi.ac.uk/interpro/) in the SwissProt entry of the respective gene.
(0.03 MB PDF)

Table S2 Cellular fatty acid patterns of *Congregibacter litoralis* KT71^T^ and *Haliea salexigens* 3X/A02/235^T^. Strain KT71^T^ was incubated for 5 to 7 days at 28°C in defined medium containing malate as substrate or MB medium (DIFCO 2216) under dim light at the gas atmosphere indicated. Values are percentages of total fatty acids; values over 5% are in bold. Data for *Haliea salexigens* were taken from Urios et al. [13]. ^a^ Chemoheterotrophically grown unpigmented cells. ^b^ Cells were grown on agar plates. ^c^ Summed feature 7 contained one or more of the following fatty acids: 19∶1 ω6c, 19∶0 cyclo and an unknown fatty acid with an equivalent chain length of 18.846 which could not be unambiguously identified.
(0.05 MB PDF)

Figure S1 Profiles of the electron transport chain in strain KT71^T^. Redox difference spectroscopy of extracts from whole cells solubilized with LDAO (A–C) or intact cells (D). Four different growth conditions were analyzed: Unpigmented cells growing with 5 mM DL-malate as substrate (top panel), cells growing photoheterotrophically with 5 mM DL-malate as substrate (second panel), unpigmented cells growing in defined SMP medium (third panel) and unpigmented cells growing in complex SYPG medium (bottom panel). All batch cultures were incubated at an initial oxygen concentration of 6 vol% at 28°C in the light (photoheterotrophic growth) or darkness (chemoheterotrophic growth). A.U., arbitrary units of absorbance. (A) Ascorbate-reduced *minus* ferricyanide-oxidized (black line) and dithionite-reduced *minus* ferricyanide-oxidized (grey line) redox difference spectra. Bands at 445 and 601 nm indicate cytochromes *a*, the peak at 552 nm indicates *c*-type cytochromes. (B) Cyanide-treated *minus* air-oxidized (black line) and cyanide and ascorbate-reduced *minus* air-oxidized (grey line) difference spectra. The thick grey line indicates the base line before addition of cyanide. Bleaching of the spectrum around 480 nm suggests a reduction of high-potential iron-sulfur proteins. The peak at 552 nm indicates reduction of *c*-type cytochromes. (C) Stigmatellin and dithionite-reduced *minus* dithionite-reduced difference spectra. The thick grey line indicates the base line before addition of stigmatellin. A negative shoulder at 445 nm and a trough at 601 nm indicate oxidation of *a*-type cytochromes. A trough at 552 nm indicates oxidation of cytochromes *c*. (D) CO and dithionite-reduced *minus* dithionite-reduced difference spectra. Troughs in the Soret region at 428 and 445 nm indicate binding of CO by heme *b* and *aa*_3_, respectively. A trough at 552 nm and a negative shoulder at 564 nm suggest presence of CO-binding cytochromes *c* and *b*, respectively.
(0.69 MB PDF)

Figure S2 Expression of the photosynthetic apparatus in cultures growing in darkness. All cultures were incubated at 28°C and subcultured for at least five times in the same medium without illumination. Cells used as inoculum were grown photoheterotrophically in SMFC medium and displayed an average expression level of 0.85 (A880 nm/A660 nm). Different colors were used to visualize variations of the expression level of the photosynthetic apparatus.
(0.02 MB PDF)

Figure S3 Growth response in various media supplemented with fluoroacetate. Growth curves were determined in medium without fluoroacetate (filled circles), 0.4 mM fluoroacetate (open circles) and 2.0 mM fluoroacetate (open triangles). The level of pigment expression in cells growing in medium without fluoroacetate was determined as A880 nm/A660 nm values (filled squares). The red arrow indicates the point in time at which fluoroacetate was added. All cultures were incubated at 28°C with an initial oxygen concentration of 12 vol% under dim light. (A) Cultures growing in SMP medium; (B) cultures growing photoheterotrophically with 6 mM DL-malate as substrate; (C) cultures growing in SYPG medium; (D) cultures of unpigmented cells growing with 6 mM DL-malate.
(0.19 MB PDF)

Figure S4 Oxygen relationship of cells growing in deep agar cultures on various substrates. Photographs were taken 5–7 days after inoculation with a culture growing on the same substrate in liquid culture. Unless noted otherwise cells used for inoculation were unpigmented. Red arrows indicate the position of maximal cell concentration in the agar column. All semisolid cultures were incubated in dim light at 28°C using the following carbon sources or substrate mixtures: (1) 10 mM acetate; (2) 5 mM DL-malate; (3) 5 mM oxaloacetate; (4) 5 mM DL-malate (pigmented cells); (5) 3 mM pyruvate and 3 mM DL-malate (equivalent to SMP medium); (6) 5 mM pyruvate; (7) 0.50 g l^−1^ yeast extract, 0.25 g l^−1^ trypticase peptone and 0.10 g l^−1^ sodium L-glutamate (equivalent to SYPG medium); (8) 2 mM sucrose.
(0.30 MB PDF)

Figure S5 Possible control of the expression of photosynthesis genes by the transcriptional regulator PpsR. (A) Arrangement of genes and putative PpsR binding sites. Green, *bch* genes; red, *puf* genes; orange, *crt* genes; blue, *hem* genes; purple, genes for sensor proteins. Red vertical arrows indicate the location of single palindromic sequences (18-mers) that could mediate the binding of PpsR. Regions of the PGS that could be transcribed into polycistronic messenger RNA are denoted by turquoise horizontal arrows. Note the clustering of PpsR binding sites in the upstream regions of both putative polycistronic transcripts. (B) Table illustrating the alignment of PpsR binding sites with the consensus sequence (shown in the first line). The orientation of the PpsR sequence motif between adjacent genes is indicated by horizontal arrows and numbers. Negative numbers indicate a distance in nucleotides to the start codon of the downstream gene. Positive numbers indicate the distance in nucleotides to the stop codon of the flanking upstream gene. Horizontal arrows indicate the direction of transcription. Nucleotides that differ from the conserved PpsR binding site (highlighted in yellow) are shown in red.
(0.09 MB PDF)
