# Supplementary material for: The Photosynthetic Apparatus and Its Regulation in the Aerobic Gammaproteobacterium Congregibacter litoralis gen. nov., sp. nov
Source: PLoS One. 2009 Mar 16;4(3):e4866. doi: 10.1371/journal.pone.0004866 (PMC2654016; doi:10.1371/journal.pone.0004866)
Supplement: Table S1 — Relevant genes encoding proteins of the KT71T electron transport chain. ORF, open reading frame; MW, molecular weight in Dalton; pI, isoelectric point. a Designation of the putatively expressed protein with the proposed gene abbreviation in parentheses. b Designation of functional sites is based on domain annotation given by INTERPRO (http://www.ebi.ac.uk/interpro/) in the SwissProt entry of the respective gene. (0.03 MB PDF) [file pone.0004866.s001.pdf]

Table S1.

Annotation [a]	ORF name	MW	pI	Functional sites [b]
Periplasmic cytochromes c:				
Cytochrome c'	KT71_16776	17827	6.11	monoheme *c*
Cytochrome c4 (*cycA1*)	KT71_07744	24968	7.66	diheme *c*
Cytochrome c4 (*cycA2*)	KT71_04580	22132	6.31	diheme *c*
Cytochrome c4 (*cycA3*)	KT71_01590	22954	4.99	diheme *c*
Cytochrome c5 (*cycB1*)	KT71_07009	14048	4.17	monoheme *c*
Cytochrome c5 (*cycB2*)	KT71_04585	10269	5.63	monoheme *c*
High-potential iron-sulfur proteins:				
High-potential iron-sulfur protein (*hpiA*)	KT71_06222	11252	8.39	TAT signal sequence<br>[4Fe-4S] cluster
High-potential iron-sulfur protein (*hpiB*)	KT71_07824	20933	4.51	[4Fe-4S] cluster
Photosynthetic reaction center:				
Reaction center cytochrome c subunit (*pufC*)	KT71_19428	39321	5.80	signal peptide<br>lipid attachment site<br>tetraheme *c*
Reaction center protein M (*pufM*)	KT71_19433	36511	6.65	
Reaction center protein L (*pufL*)	KT71_19438	32695	5.79	
Reaction center protein H (*puhA*)	KT71_19393	25345	4.84	
Cytochrome c reductase:				
Rieske Fe-S protein (*petA*)	KT71_10307	21703	5.43	TAT signal sequence<br>[2Fe-2S] cluster
Cytochrome bc_1 subunit (*petBC*)	KT71_10312	75933	5.50	2 heme *b*<br>monoheme *c*
Terminal oxidases:				
***cbb_3*-type 1**				
Subunit I (*ccoN1*)	KT71_16991	53294	9.04	Cu_B<br>2 heme *b*
Subunit II (*ccoO1*)	KT71_16996	22665	6.31	monoheme *c*
Subunit III (*ccoP1*)	KT71_17006	32268	5.25	diheme *c*
Subunit IV (*ccoQ*)	KT71_17001	6572	4.42	
***cbb_3*-type 2**				
Subunit I (*ccoN2*)	KT71_06177	60531	6.49	Cu_B<br>2 heme *b*
Subunit II (*ccoO2*)	KT71_06182	22203	4.64	monoheme *c*
Subunit III (*ccoP2*)	KT71_06187	21261	5.22	monoheme *c*
***caa_3*-type**				
Subunit I (*ctaD*)	KT71_04635	58204	6.25	Cu_B<br>2 heme *a*
Subunit II (*ctaC*)	KT71_04640	43217	4.69	Cu_A<br>monoheme *c*
Subunit III (*ctaE*)	KT71_04625	36239	5.90	phospholipid binding site
