# Supplementary material for: The Photosynthetic Apparatus and Its Regulation in the Aerobic Gammaproteobacterium Congregibacter litoralis gen. nov., sp. nov
Source: PLoS One. 2009 Mar 16;4(3):e4866. doi: 10.1371/journal.pone.0004866 (PMC2654016; doi:10.1371/journal.pone.0004866)
Supplement: Table S2 — Cellular fatty acid patterns of Congregibacter litoralis KT71T and Haliea salexigens 3X/A02/235T. Strain KT71T was incubated for 5 to 7 days at 28°C in defined medium containing malate as substrate or MB medium (DIFCO 2216) under dim light at the gas atmosphere indicated. Values are percentages of total fatty acids; values over 5% are in bold. Data for Haliea salexigens were taken from Urios et al. [13]. a Chemoheterotrophically grown unpigmented cells. b Cells were grown on agar plates. c Summed feature 7 contained one or more of the following fatty acids: 19∶1 ω6c, 19∶0 cyclo and an unknown fatty acid with an equivalent chain length of 18.846 which could not be unambiguously identified. (0.05 MB PDF) [file pone.0004866.s002.pdf]

Table S2.

Fatty acid	3X/A02/235[T]	KT 71[T] (MB DIFCO 2216)			KT 71[T] (6 mM DL-malate)[a]		
		Air[b]	O_2< 12%	O_2< 6%	O_2< 21%	O_2< 12%	O_2< 6%
Saturated fatty acids							
10:0	-	0.5	0.5	0.5	0.4	0.4	0.5
11:0	1.0	1.0	-	-	-	-	-
12:0	1.6	2.2	2.6	2.8	3.1	2.5	2.6
13:0	1.3	1.0	-	-	-	-	-
14:0	1.3	2.0	3.3	3.3	2.8	2.8	2.8
15:0	4.5	4.9	0.5	0.6	-	0.2	-
16:0	2.0	**5.4**	**9.0**	**9.6**	**15.9**	**17.3**	**15.7**
16:0 N alcohol	-	-	0.7	1.2	-	-	-
17:0	**9.3**	3.1	0.3	0.5	-	0.4	0.6
17:0 10 methyl	-	-	0.9	1.5	-	0.8	1.1
18:0	-	0.6	0.7	1.1	1.5	1.6	2.0
**Sum**	**21.0**	**20.7**	**18.5**	**21.1**	**23.7**	**26.0**	**25.3**
Unsaturated fatty acids							
15:1 ω6c	**5.8**	2.0	-	-	-	-	-
15:1 ω8c	-	0.3	-	-	-	-	-
16:1 ω6c	-	**6.5**	**26.7**	**29.0**	**6.0**	**12.9**	**12.0**
16:1 ω7c	**21.2**	**23.1**	**16.1**	**13.0**	**27.5**	**18.1**	**20.4**
16:1 ω7c alcohol	-	0.2	0.5	-	-	-	-
17:1 ω6c	2.7	2.8	-	-	-	-	-
17:1 ω8c	**23.9**	**8.1**	0.4	0.5	-	-	-
18:1 ω5c	-	0.1	-	-	-	-	-
18:1 ω6c	-	-	**14.8**	**17.0**	-	-	-
18:1 ω7c	**17.5**	**29.7**	**17.3**	**14.7**	**37.8**	**39.3**	**37.8**
11 methyl 18:1 ω7c	-	-	1.4	1.4	-	-	1.1
18:1 ω9c	-	-	0.8	0.6	-	-	-
**Sum**	**71.1**	**72.8**	**78.0**	**76.2**	**71.3**	**70.3**	**71.3**
Hydroxy fatty acids							
9:0 3OH	-	0.2	-	-	-	-	-
10:0 3OH	1.8	2.1	2.4	2.1	2.7	2.0	1.8
11:0 3OH	3.3	0.5	-	-	-	-	-
12:0 3OH	1.1	0.4	1.1	1.0	1.2	1.2	1.0
11:0 ISO 3OH	3.3	0.1	-	-	-	-	-
16:0 ISO 3OH	-	-	-	-	-	-	-
**Sum**	**9.5**	3.3	3.5	3.1	3.9	3.2	2.8
Sum in Feature 7[c]	-	2.8	-	-	1.1	0.6	0.4
