# Supplementary figures and images for: The Photosynthetic Apparatus and Its Regulation in the Aerobic Gammaproteobacterium Congregibacter litoralis gen. nov., sp. nov
Source: PLoS One. 2009 Mar 16;4(3):e4866. doi: 10.1371/journal.pone.0004866 (PMC2654016; doi:10.1371/journal.pone.0004866)

Figure S1.

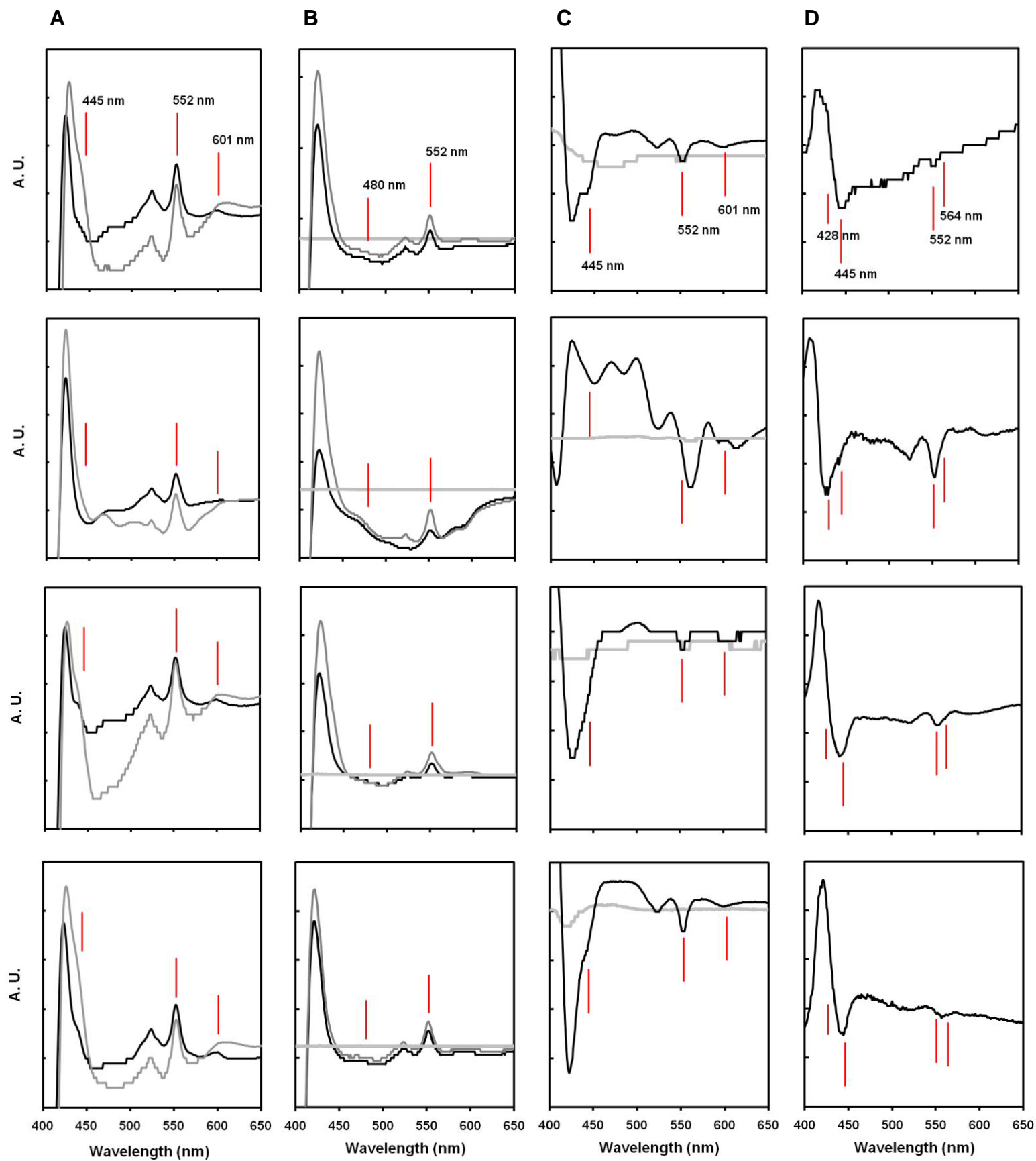

Supplement: Figure S1 — Profiles of the electron transport chain in strain KT71T. Redox difference spectroscopy of extracts from whole cells solubilized with LDAO (A–C) or intact cells (D). Four different growth conditions were analyzed: Unpigmented cells growing with 5 mM DL-malate as substrate (top panel), cells growing photoheterotrophically with 5 mM DL-malate as substrate (second panel), unpigmented cells growing in defined SMP medium (third panel) and unpigmented cells growing in complex SYPG medium (bottom panel). All batch cultures were incubated at an initial oxygen concentration of 6 vol% at 28°C in the light (photoheterotrophic growth) or darkness (chemoheterotrophic growth). A.U., arbitrary units of absorbance. (A) Ascorbate-reduced minus ferricyanide-oxidized (black line) and dithionite-reduced minus ferricyanide-oxidized (grey line) redox difference spectra. Bands at 445 and 601 nm indicate cytochromes a, the peak at 552 nm indicates c-type cytochromes. (B) Cyanide-treated minus air-oxidized (black line) and cyanide and ascorbate-reduced minus air-oxidized (grey line) difference spectra. The thick grey line indicates the base line before addition of cyanide. Bleaching of the spectrum around 480 nm suggests a reduction of high-potential iron-sulfur proteins. The peak at 552 nm indicates reduction of c-type cytochromes. (C) Stigmatellin and dithionite-reduced minus dithionite-reduced difference spectra. The thick grey line indicates the base line before addition of stigmatellin. A negative shoulder at 445 nm and a trough at 601 nm indicate oxidation of a-type cytochromes. A trough at 552 nm indicates oxidation of cytochromes c. (D) CO and dithionite-reduced minus dithionite-reduced difference spectra. Troughs in the Soret region at 428 and 445 nm indicate binding of CO by heme b and aa 3, respectively. A trough at 552 nm and a negative shoulder at 564 nm suggest presence of CO-binding cytochromes c and b, respectively. (0.69 MB PDF) [file pone.0004866.s003.pdf]

Figure S3.

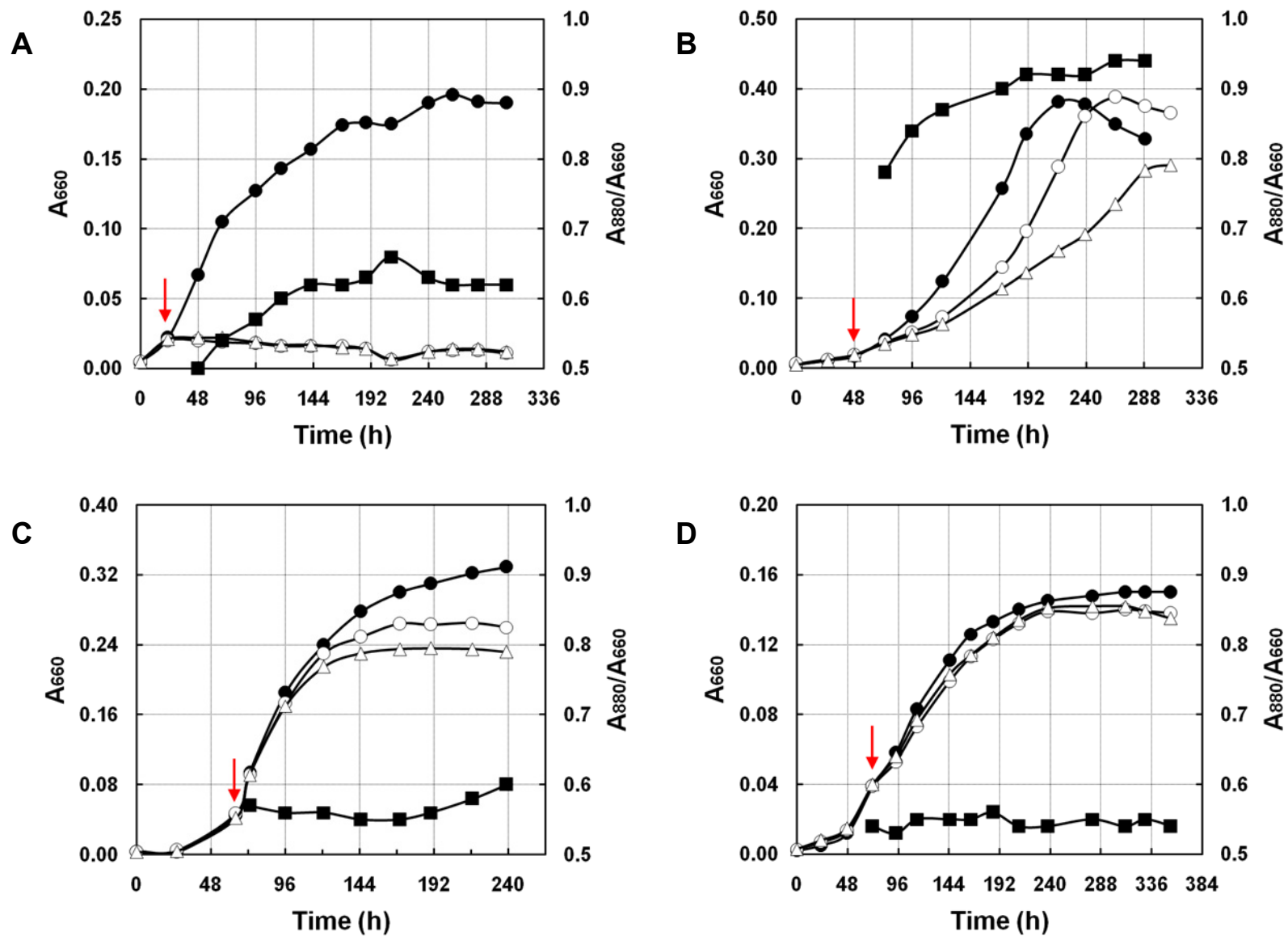

Supplement: Figure S3 — Growth response in various media supplemented with fluoroacetate. Growth curves were determined in medium without fluoroacetate (filled circles), 0.4 mM fluoroacetate (open circles) and 2.0 mM fluoroacetate (open triangles). The level of pigment expression in cells growing in medium without fluoroacetate was determined as A880 nm/A660 nm values (filled squares). The red arrow indicates the point in time at which fluoroacetate was added. All cultures were incubated at 28°C with an initial oxygen concentration of 12 vol% under dim light. (A) Cultures growing in SMP medium; (B) cultures growing photoheterotrophically with 6 mM DL-malate as substrate; (C) cultures growing in SYPG medium; (D) cultures of unpigmented cells growing with 6 mM DL-malate. (0.19 MB PDF) [file pone.0004866.s005.pdf]

**Figure S4.**

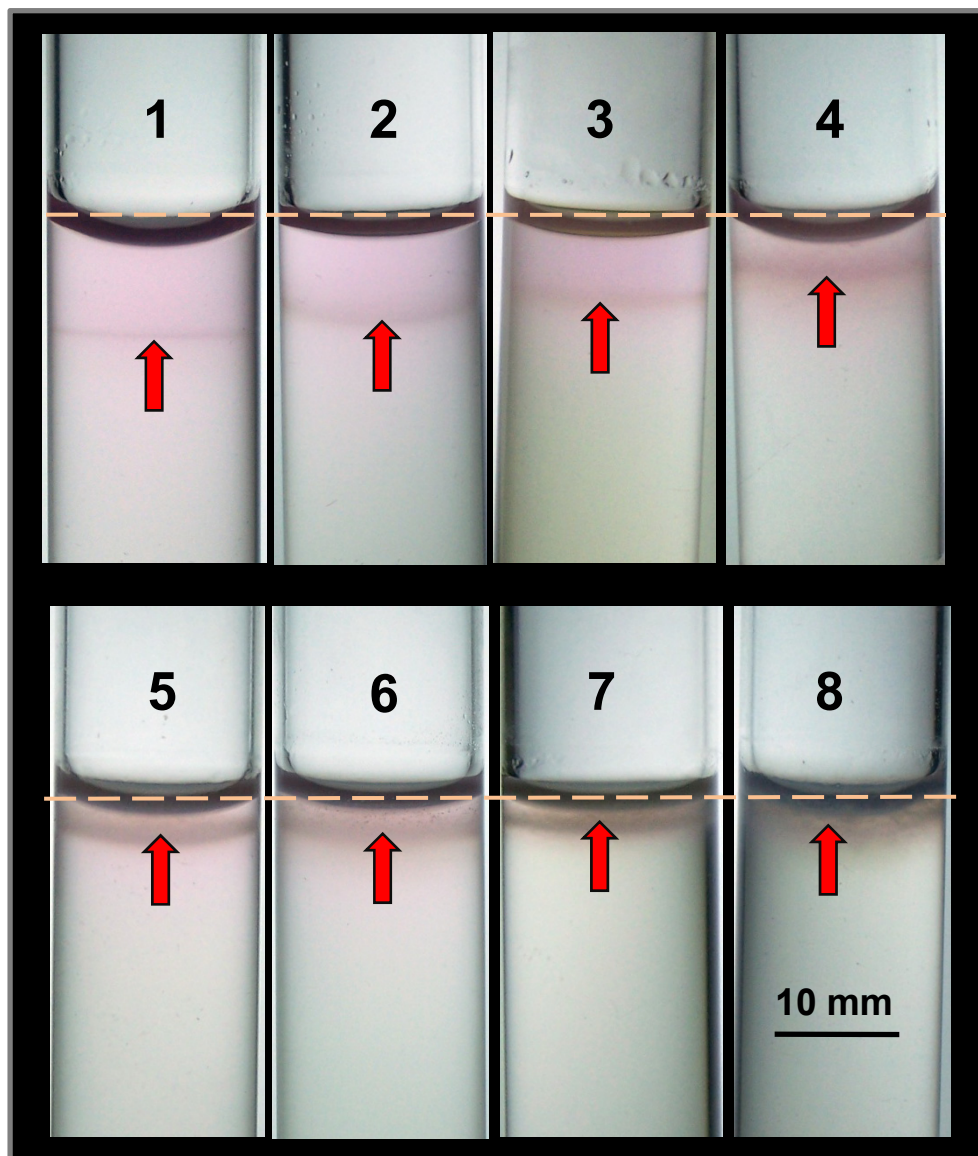

Supplement: Figure S4 — Oxygen relationship of cells growing in deep agar cultures on various substrates. Photographs were taken 5–7 days after inoculation with a culture growing on the same substrate in liquid culture. Unless noted otherwise cells used for inoculation were unpigmented. Red arrows indicate the position of maximal cell concentration in the agar column. All semisolid cultures were incubated in dim light at 28°C using the following carbon sources or substrate mixtures: (1) 10 mM acetate; (2) 5 mM DL-malate; (3) 5 mM oxaloacetate; (4) 5 mM DL-malate (pigmented cells); (5) 3 mM pyruvate and 3 mM DL-malate (equivalent to SMP medium); (6) 5 mM pyruvate; (7) 0.50 g l−1 yeast extract, 0.25 g l−1 trypticase peptone and 0.10 g l−1 sodium L-glutamate (equivalent to SYPG medium); (8) 2 mM sucrose. (0.30 MB PDF) [file pone.0004866.s006.pdf]
