# Supplementary material for: The Photosynthetic Apparatus and Its Regulation in the Aerobic Gammaproteobacterium Congregibacter litoralis gen. nov., sp. nov
Source: PLoS One. 2009 Mar 16;4(3):e4866. doi: 10.1371/journal.pone.0004866 (PMC2654016; doi:10.1371/journal.pone.0004866)
Supplement: Figure S2 — Expression of the photosynthetic apparatus in cultures growing in darkness. All cultures were incubated at 28°C and subcultured for at least five times in the same medium without illumination. Cells used as inoculum were grown photoheterotrophically in SMFC medium and displayed an average expression level of 0.85 (A880 nm/A660 nm). Different colors were used to visualize variations of the expression level of the photosynthetic apparatus. (0.02 MB PDF) [file pone.0004866.s004.pdf]

Figure S2.

Substrate	Number of Transfers				
	1	2	3	4	5
2 mM Fumarate	0.91	0.86	0.83	0.83	0.80
2 mM Succinate	0.90	0.85	0.78	0.82	0.80
4 mM DL-Malate	0.90	0.85	0.84	0.84	0.67
4 mM DL-Malate + 100 μM FeCitrate	0.79	0.79	0.78	0.78	0.71
2 mM 2-Oxoglutarate (A)	0.85	0.78	0.81	0.70	0.78
2 mM 2-Oxoglutarate (B)	0.79	0.60	0.55	0.54	0.55
2 mM Propionate	0.74	0.60	0.54	0.53	0.53
2 mM Oxaloacetate	0.56	0.53	0.57	0.52	0.57
2 mM Pyruvate	0.56	0.53	0.53	0.53	0.53
