# Supplementary material for: The Photosynthetic Apparatus and Its Regulation in the Aerobic Gammaproteobacterium Congregibacter litoralis gen. nov., sp. nov
Source: PLoS One. 2009 Mar 16;4(3):e4866. doi: 10.1371/journal.pone.0004866 (PMC2654016; doi:10.1371/journal.pone.0004866)
Supplement: Figure S5 — Possible control of the expression of photosynthesis genes by the transcriptional regulator PpsR. (A) Arrangement of genes and putative PpsR binding sites. Green, bch genes; red, puf genes; orange, crt genes; blue, hem genes; purple, genes for sensor proteins. Red vertical arrows indicate the location of single palindromic sequences (18-mers) that could mediate the binding of PpsR. Regions of the PGS that could be transcribed into polycistronic messenger RNA are denoted by turquoise horizontal arrows. Note the clustering of PpsR binding sites in the upstream regions of both putative polycistronic transcripts. (B) Table illustrating the alignment of PpsR binding sites with the consensus sequence (shown in the first line). The orientation of the PpsR sequence motif between adjacent genes is indicated by horizontal arrows and numbers. Negative numbers indicate a distance in nucleotides to the start codon of the downstream gene. Positive numbers indicate the distance in nucleotides to the stop codon of the flanking upstream gene. Horizontal arrows indicate the direction of transcription. Nucleotides that differ from the conserved PpsR binding site (highlighted in yellow) are shown in red. (0.09 MB PDF) [file pone.0004866.s007.pdf]

Figure S5.

A

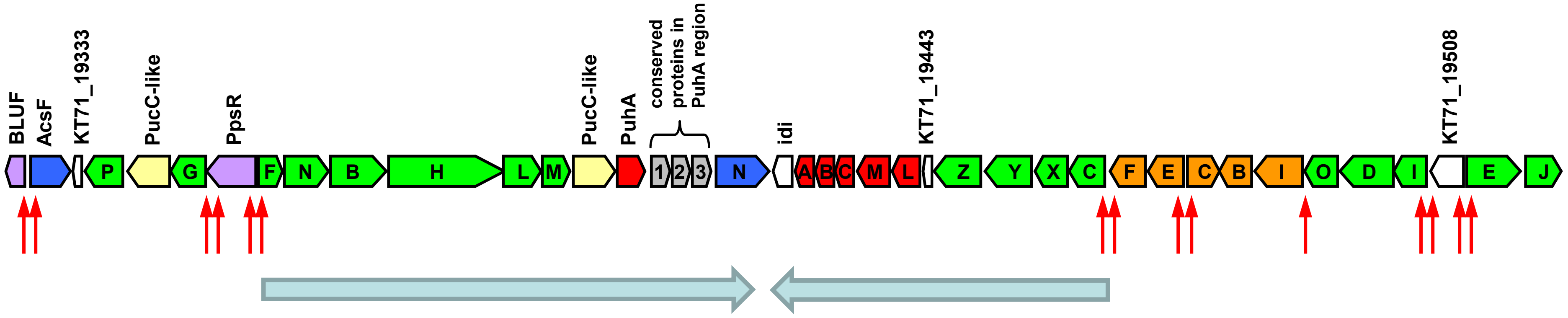

B

TGTcaNNNNNNNNtgACA -N(8)- TGTcaNNNNNNNNtgACA						
BLUF	- 246	←	TGTCAACTAAACTTGACTAT.TATATTGTCAAGAATAGTTAACT	→	- 65	*acsF*
bchG	- 4	←	CGTCAATTAAAATTGACACTATAAAGTGTCGGTTCTATATTACA	←	+ 83	*ppsR*
ppsR	- 176	←	TGTCAATTATAATTTACACATTGATGCGTGTAAAAGAATTGACA	→	- 148	*bchF*
bchC	- 43	←	TGTCATTTTTGCTTGACAGCAGTTGCTGTCCATATAAACTGACA	←	+ 26	*crtF*
crtE	- 15	←	CGTCTAATCTACCTTACACCATTAAGTGTAAACTAATATTAACA	→	- 44	*crtC*
crtI	- 18	←	TGTAAAGCAAGTTTGACA	←	+ 53	*bchO*
bchI	- 48	←	TCTCATCGGCTAATGACA -N(20)- TGTAAGCTTGGATTTACA	←	+ 27	KT71_19508
KT71_19508	- 179	←	TGTCCAATAAATGTAACACC.TAAAGTGTTAACTAAATTTGACA	→	-21	*bchE*
